# Overexpression of aberrant Wnt5a and its effect on acquisition of malignant phenotypes in adult T-cell leukemia/lymphoma (ATL) cells

**DOI:** 10.1038/s41598-021-83613-2

**Published:** 2021-02-18

**Authors:** Kazumi Nakano, Yohei Chihara, Seiichiro Kobayashi, Masako Iwanaga, Atae Utsunomiya, Toshiki Watanabe, Kaoru Uchimaru

**Affiliations:** 1 Department of Computational Biology and Medical Sciences, Graduate School of Frontier Sciences, The University of Tokyo, 4-6-1, Shirokanedai, Minatoku, Tokyo, 108-8639 Japan; 2 Department of Hematology, Kanto Rosai Hospital, Kanagawa, Japan; 3 Department of Clinical Epidemiology, Graduate School of Biomedical Science, Nagasaki University, Nagasaki, Japan; 4 Department of Hematology, Imamura General Hospital, Kagoshima, Japan; 5 Laboratory of Practical Management of Medical Information, Graduate School of Medicine, St. Marianna University, Kanagawa, Japan

**Keywords:** Haematological cancer, Oncogenes, Tumour biomarkers, Tumour virus infections, Cell growth, Cell migration, Transcription, Cancer, Cell biology, Molecular biology, Oncology, HTLV, Retrovirus, Viral pathogenesis, Microbiology, Virology

## Abstract

Wnt5a is a ligand of the non-canonical Wnt signaling pathway involved in cell differentiation, motility, and inflammatory response. Adult T-cell leukemia/lymphoma (ATL) is one of the most aggressive T-cell malignancies caused by infection of human T-cell leukemia virus type1 (HTLV-1). Among subtypes of ATL, acute-type ATL cells are particularly resistant to current multidrug chemotherapies and show remarkably high cell-proliferative and invasive phenotypes. Here we show a dramatic increase of *WNT5A* gene expression in acute-type ATL cells compared with those of indolent-type ATL cells. Treatment with IWP-2 or Wnt5a-specific knockdown significantly suppressed cell growth of ATL-derived T-cell lines. We demonstrated that the overexpression of c-Myb and FoxM1 was responsible for the synergistic activation of the *WNT5A* promoter. Also, a *WNT5A* transcript variant without the exon4 (the *ΔE4-WNT5A* mRNA), encoding ΔC-Wnt5 (1-136aa of 380aa), is overexpressed in acute-type ATL cells. The ΔC-Wnt5a is secreted extracellularly and enhances cellular migration/invasion to a greater extent compared with wildtype (WT)-Wnt5a. Moreover, the ΔC-Wnt5a secretion was not suppressed by IWP-2, indicating that this mutant Wnt5a is secreted via a different pathway from the WT-Wnt5a. Taken together, synergistic overexpression of the ΔC-Wnt5a by c-Myb and FoxM1 may be responsible for the malignant phenotype of acute-type ATL cells.

## Introduction

Adult T-cell leukemia/lymphoma (ATL) is a T-cell malignancy caused by infection of human T-cell leukemia virus type 1 (HTLV-1) with CD4^+^ T cells^1^. The main route of infection is mother-to-child transmission via breast milk, and it is estimated that there are approximately 5–10 million or more HTLV-1 carriers world-wide^2–4^. ATL is developed in about 5% of carriers after a long chronic infection period of 50–60 years, with the average onset-age of 60 years old. ATL is classified into the indolent type (including the smoldering and the chronic types), and the aggressive type (including the acute and the lymphoma types), and the prognosis varies depending on the type of disease^5^. A retrospective analysis of the long-term prognosis of indolent-type ATL patients showed a 5-year survival rate of 47.2%. Aggressive-type ATL, on the other hand, shows poor prognosis with a median overall survival of 6–10 months and a 3-year overall survival rate of 24%, without effective treatments^6^. The aggressive-type ATL cells are characterized by high cell-proliferative and invasive ability that causes uncontrollable metastasis to various organs. Still, the factors responsible for acquiring the malignant phenotypes have not been identified.

The Wnt pathway is a highly conserved signal pathway in eukaryotes and is known to be involved in various biological responses such as developmental differentiation, angiogenesis, and cell motility^7^. The Wnt pathway is a complex pathway involving 19 types of Wnt family proteins, 10 types of Frizzled (Fz) as receptors, and LRP5, LRP6, Ror1, Ror2, Ryk are known as their coupled receptors^8^. The Wnt pathway is divided into the canonical and noncanonical pathways depending on the β-catenin-dependent or not, respectively. The canonical pathway is mainly activated by Wnt3a in the cell in a β-catenin-dependent manner and is involved primarily in cell proliferation and differentiation^9^. On the other hand, Wnt5a activates the non-canonical pathway, which regulates cell movement by actin polymerization via a planar cell polarity pathway and a Ca^2+^-dependent pathway in a β-catenin-independent manner^10–14^. The planar cell polarity pathway regulates the activities of Rho/Rock and Rac/JNK (Jun-N-terminal kinase) via Wnt receptor Fz and cytoplasmic binding of Dishevelled (Dvl) to Fz promotes cell movement through actin polymerization. The intracellular Ca^2+^-concentration is increased in the Ca^2+^-dependent pathway, which activates PKC and CaMK to promote actin polymerization through activation of CDC42. PKC and CaMK are expected to suppress β-catenin. The balance between the canonical and non-canonical Wnt activities is known to regulate stem-cell self-renewal/differentiation and aging^15,16^.

To date, abnormal activation of the β-catenin-dependent Wnt pathway has been reported to be associated with cell carcinogenesis, particularly in solid tumors^9,17–19^. Overexpression of Wnt5a has been reported to promote the invasive and metastatic characters of tumor cells^20–22^. The expression of Wnt3a is suppressed in HTLV-1-infected cells and ATL cells. In contrast, high expression of Wnt5a promotes differentiation of osteoclast precursor cells into osteoclasts, which may induce hypercalcemia, a typical symptom of ATL^23^. It has also been reported that the HTLV-1 viral protein Hbz (HTLV-1 bZIP factor), which is encoded by the antisense HTLV-1 genome, regulates the expression of Wnt5a in HTLV-1-infected cells^24^. Wnt5a is thus attracting attention as a useful therapeutic target for ATL. Still, the differences in the expression pattern of the *WNT5A* gene among disease-subtypes of ATL have not been clearly described. Also, the detailed molecular mechanism of its overexpression and effects in ATL cells are still waiting to be elucidated.

## Results

### Wnt5a overexpression in ATL cells

**Figure 1:**
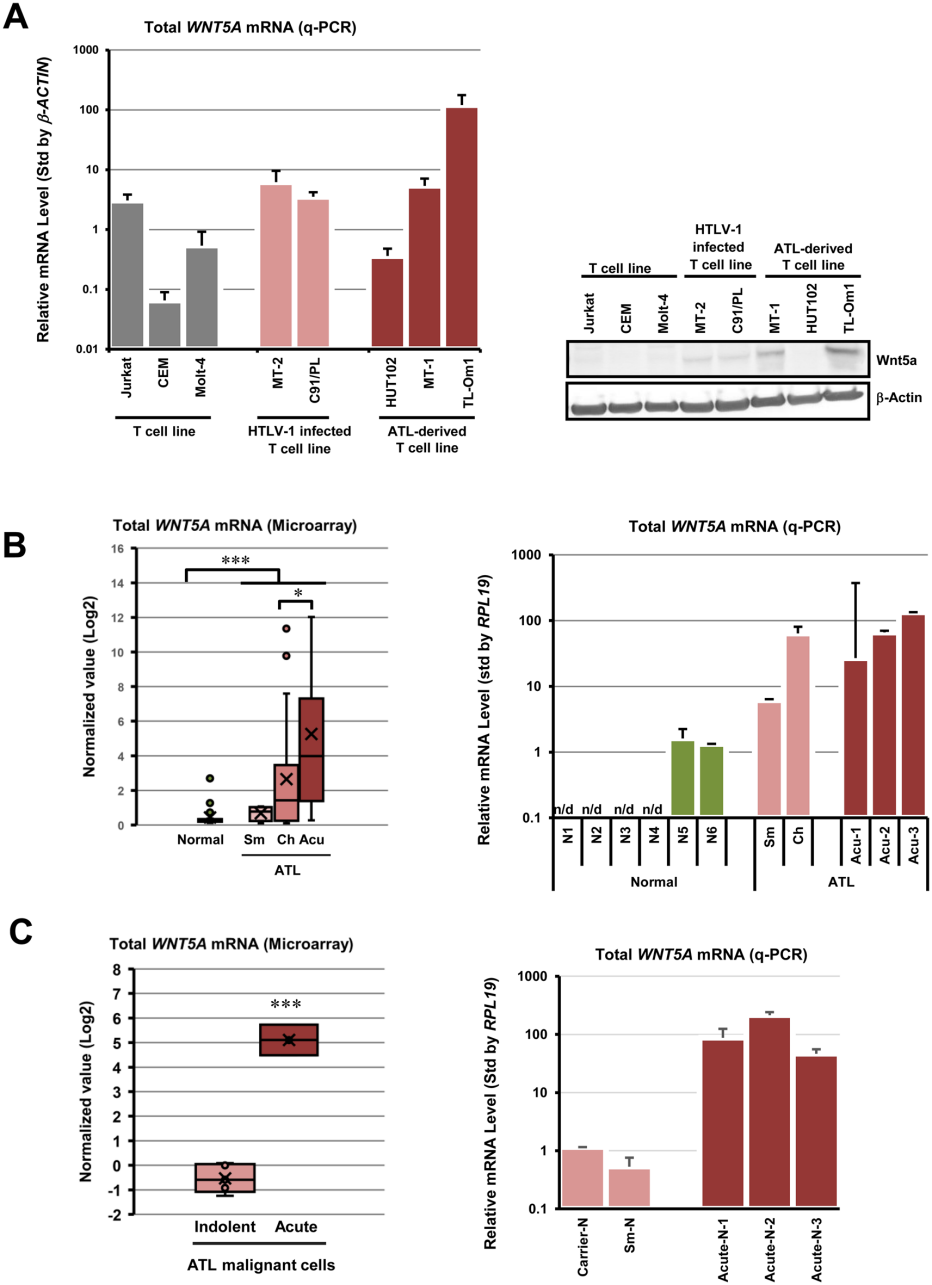
Overexpression of Wnt5a in ATL cells. (**A**) The *WNT5A* mRNA levels were examined in various cell lines. Compared with HTLV-1 uninfected cell lines (Jurkat, CEM, and Molt-4), HTLV-1 infected cell lines (MT-2 and C91/PL) and ATL patient-derived cell lines (HUT102, MT-1, and TL-Om1) show 10 to 100-folds higher *WNT5A* mRNA levels (left-hand side graph). A similar tendency was also observed at the Wnt5a protein level. Especially, TL-Om1 showed the highest mRNA and protein expression levels of the *WNT5A* gene. Please note that the right-hand side panel consists of two pictures from two separated blots for Wnt5a and β-Actin, respectively, which are clearly separated by black frames. (**B**) The total *WNT5A* mRNA levels were re-analyzed in gene expression profiling analysis (left-hand side graph, GSE33615, PBMCs from ATL patients n = 52 and normal CD4^+^ T cells n = 21, *P < 0.05; ***P < 0.001) and were confirmed in the quantitative-PCR analysis (right-hand side graph, PBMCs from ATL patients n = 5 and normal CD4^+^ T cells n = 6). Both data demonstrated that the *WNT5A* mRNA level increased up to 100-folds in PBMCs from ATL patients compared with normal CD4^+^ T cells. In gene expression profiling data, the *WNT5A* mRNA level was significantly higher in PBMCs from acute-type ATL patients than indolent-type ATL patients. (**C**) The total *WNT5A* mRNA levels were examined in primary malignant ATL cells in gene expression profiling analysis (left-hand side graph, GSE55851, Indolent-type ATL n = 5 and acute-type ATL n = 3, ***P < 0.001) and in quantitative-PCR analysis (right-hand side graph, asymptomatic carrier n = 1, indolent-type ATL n = 1, and acute-type ATL n = 3). In both experiments, malignant ATL cells were isolated as CADM1 + /CD7^−^ CD4^+^ T cells^26^. Both graphs show that the expression level of the total *WNT5A* mRNA increases about 100-folds in acute-type cells compared with that in indolent-type ATL cells.

As shown in Fig. 1A, we first found overexpression of *WNT5A* mRNA and Wnt5a protein in HTLV-1-related T cell lines. Most of *WNT5A* mRNA levels were more than 10 times higher in HTLV-1 infected immortal cell lines (MT-2 and C91/PL), and ATL patient-derived T-cell lines (HUT102, MT-1, and TL-Om1) compared to HTLV-1 non-infected T-cell lines (Jurkat, CEM, and Molt4). A similar tendency was observed at the Wnt5a protein level. In particular, TL-Om1 cells showed the highest *WNT5A* mRNA and Wnt5a protein levels.

Next, we determined the expression level of the total *WNT5A* mRNA in ATL patients' primary PBMC by using the data of gene expression profiling analysis in ATL cells (n = 52) and normal CD4^+^ T cells (n = 21) (GSE33615)^25^, which we reported previously. As a result, total *WNT5A* mRNA levels were increased significantly in ATL cells compared with healthy CD4^+^ T cells. Importantly, the *WNT5A* mRNA levels were increased in progression dependent manner. This result was also reproduced by q-PCR of the total *WNT5A* mRNA in primary cells (Fig. 1B).

The HAS-Flow method reported by Kobayashi et al.^26^ allows us to isolate non-infected and some infected cells, infected cells, and malignant cells of CD4^+^ T cells from individual ATL patient dependent on the expression levels of CADM1 and CD7. We conducted gene expression profiling in each cell-population (GSE55851)^26^, and further demonstrated that expression levels of the total *WNT5A* mRNA increased drastically in acute-type malignant cells compared with that in indolent-type ATL malignant cells (Fig. 1C). Also, we measured total *WNT5A* mRNA levels by qPCR in the tumor cell fraction (CD4 + /CD7 − /CADM1 +) of an HTLV-1 carrier, a smoldering-type ATL patient, and 3 acute-type ATL patients, which were concentrated by the HAS-Flow method. As a result, the total *WNT5A* mRNA expression increased more than 100-fold in the acute-type ATL tumor cells compared to the carrier HTLV-1 infected cells and smoldering-type ATL cells (Fig. 1C).

### Influence of Wnt5a on cell growth

**Figure 2:**
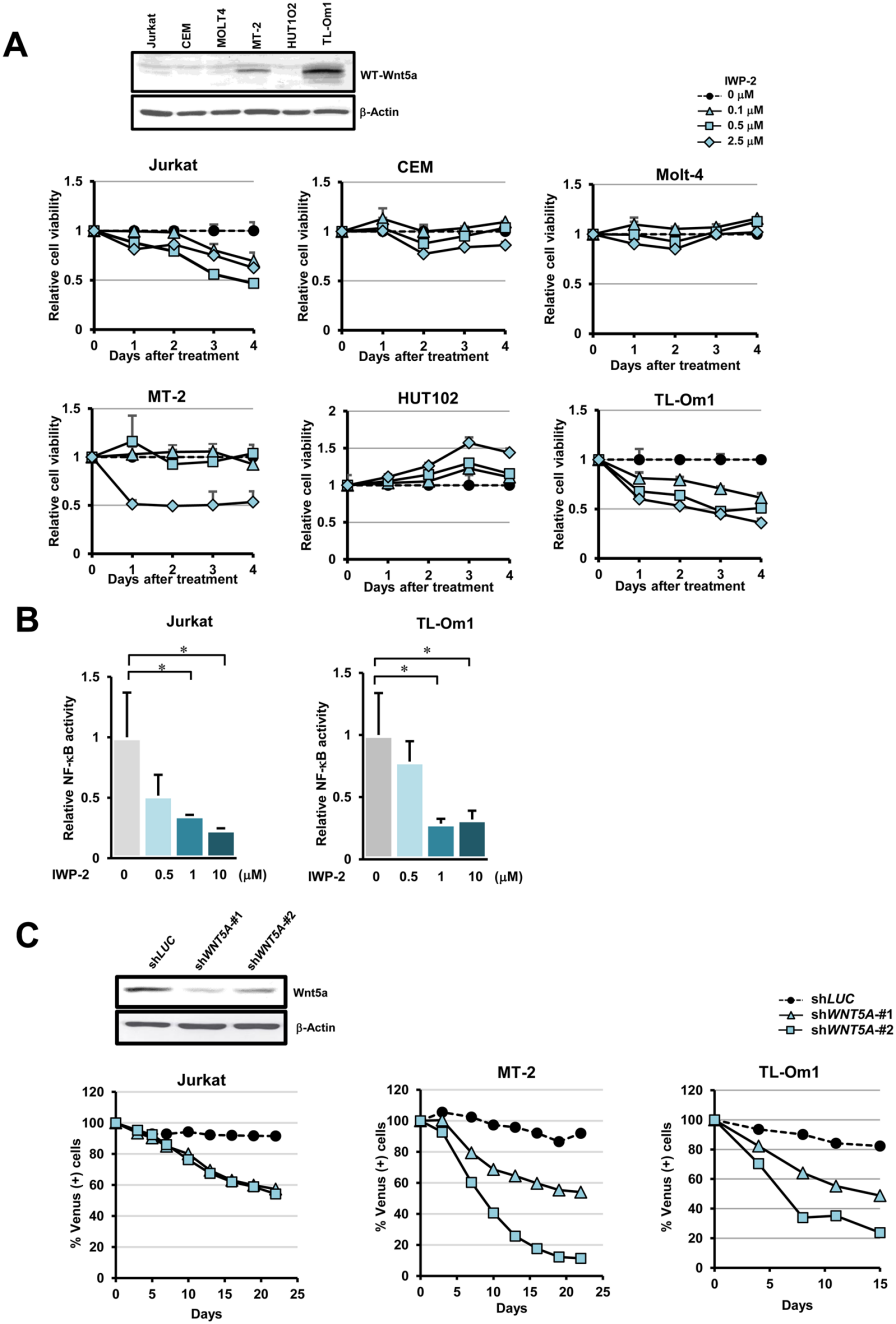
Wnt5a dependent NF-κB activation and cell growth. (**A**) IWP-2 treatment in Jurkat, CEM, Molt-4, MT-2, HUT102, and TL-Om1 cells. The dose-dependent suppression of cell growth was observed in MT-2 and TL-Om1, which expressed high levels of Wn5a protein (the upper panel). Please note that the upper panel consists of two pictures from two separated blots for Wnt5a and β-Actin, respectively, which are clearly separated by black frames. (**B**) IWP-2 treatment significantly and dose-dependently suppressed cellular NF-κB activities in Jurkat and TL-Om1 cells (n = 6, mean ± SD, *P < 0.05). (**C**) Specific knockdown of Wnt5a expression (the upper panel) by shRNA against *WNT5A* mRNA in Jurkat, MT-2, and TL-Om1 significantly reduced the cellular survival rates. Please note that the upper panel consists of two pictures from two separated blots for Wnt5a and β-Actin, respectively, which are clearly separated by black frames. The graphs show the course of changes in the percentage of Venus (+) cells, i.e., Wnt5a knockdown cells, after the recombinant lentivirus infection. The % Venus (+) cells in sh*WNT5A* expressing cells decline rapidly during infection, while that with sh*LUC* (the negative control) does not. The induction of cell-death in Wnt5a knockdown cells were more significant in MT-2 and TL-Om1 cells than Jurkat, in agreement with IWP-2 treatments in (**A**).

The effect of IWP-2, an inhibitor of Wnt secretion, on cell proliferation ability was examined in the HTLV-1 uninfected T-cell lines (Jurkat, CEM, and Molt4) and HTLV-1-related T-cell lines (MT-2, HUT1O2, and TL-Om1). As a result, cell growth was markedly suppressed in MT-2 and TL-Om1 with high Wnt5a expression levels compared to cell lines with low Wnt5a expression levels (Fig. 2A). Moreover, IWP-2 treatment significantly suppressed cellular NF-κB activity in Jurkat and TL-Om1 cells (Fig. 2B). Next, we investigated the effect of Wnt5a-specific knockdown on cell proliferation using *WNT5A*-specific shRNA. The shWnt5a encoding recombinant lentivirus transduces the shRNA together with Venus fluorescent protein (Dr. Miyoshi, RIKEN, Japan). Therefore, sh*WNT5A* expressing cells are detected as Venus (+) cells. The graphs in Fig. 2C show the course of changes in the percentage of Venus (+) cells, i.e., Wnt5a knockdown cells, after the recombinant lentivirus infection. The percentage of Venus (+) cells, i.e., sh*WNT5A* expressing cells, declines rapidly, while that expressing sh*LUC* (the negative control) does not, especially in MT-2 and TL-Om1 cells (Fig. 2C).

### Two oncogenic transcription factors, c-Myb and FoxM1, are responsible for Wnt5a overexpression

**Figure 3:**
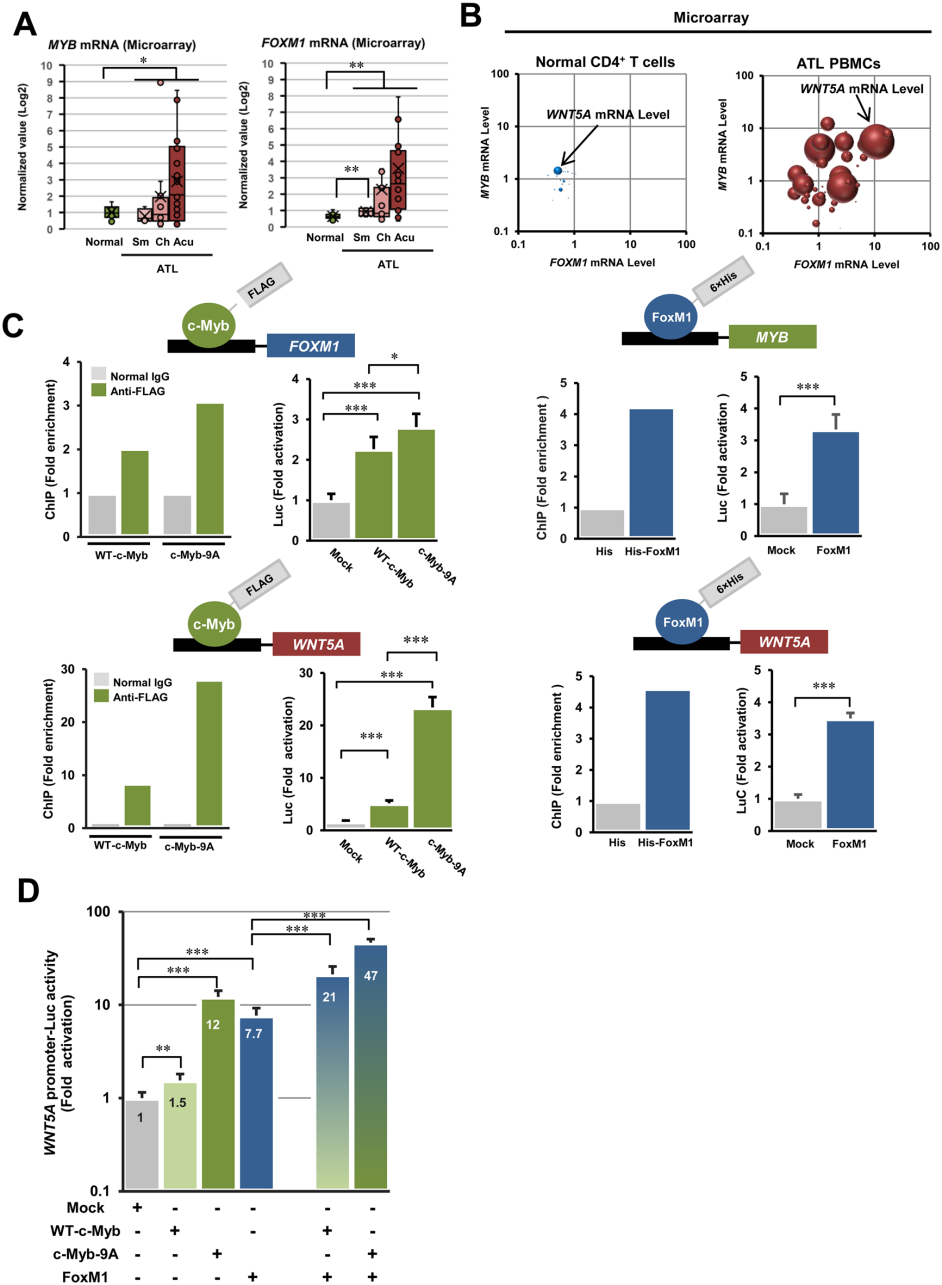
c-Myb and FoxM1 synergistically upregulate the *WNT5A* gene expression in ATL cells. (**A**) Re-analysis of the gene expression microarray data (GSE33615) show that *FOXM1* and *MYB* mRNA levels increase in ATL primary PBMCs compared with normal CD4^+^ T cells (PBMCs from ATL patients n = 52 and normal CD4^+^ T cells n = 21, *P < 0.05; **P < 0.01). (**B**) *WNT5A*, *FOXM1*, and *MYB* mRNA levels are increased in correlation in PBMCs from ATL patients (GSE33615, PBMCs from ATL patients n = 52 and normal CD4^+^ T cells n = 21). In the graphs, the x-axis shows *FOXM1* mRNA levels, the y-axis shows *MYB* mRNA levels, and the size of the bubble indicates *WNT5A* mRNA levels. Those three mRNA levels are low in Normal CD4^+^ T cells (the left-hand side graph), while they are increased altogether in ATL cells (the right-hand side panel). (**C**) The molecular relationship among Wnt5a, FoxM1, and c-Myb was investigated by ChIP assays and luciferase-based promoter activity assays (n = 6, mean ± SD, *P < 0.05; **P < 0.01; ***P < 0.001). c-Myb directly binds to the *FOXM1* promoter region and transactivates the *FOXM1* gene (the top-left), and vice versa (the top-right). Both c-Myb and FoxM1 are recruited to the promoter region of the *WNT5A* gene, and transactivate the *WNT5A* promoter (the lower graphs). The c-Myb-9A isoform, which lacks the C-terminal negative regulatory domain and shows significantly higher transactivity than the WT-c-Myb, demonstrated significantly higher recruitment and activation of *FOXM1* promoter and *WNT5A* promoter than WT-c-Myb (the top-left and the lower-left). (**D**) Simultaneous expression of FoxM1 and c-Myb elevated *WNT5A* promoter activity in a synergistic manner compared with the cases in that FoxM1 or c-Myb was expressed alone. Moreover, co-expression of the c-Myb-9A and FoxM1 showed more than twofold higher *WNT5A* transactivity compared with co-expression of the WT-c-Myb and FoxM1 (n = 6, mean ± SD, **P < 0.01; ***P < 0.001).

Upon confirming the overexpression of *WNT5A* mRNA in HTLV-1 infected cells and ATL cells, we aimed to elucidate the molecular mechanism of *WNT5A* gene overexpression in ATL cells. The analysis of GSE33615 showed that the mRNA expression levels of c-Myb and FoxM1, which are known as transcription factors that positively regulate the *WNT5A* gene, were significantly increased in PBMCs from ATL patients compared with normal CD4^+^ T cells (Fig. 3A). Figure 3B shows the expression levels of *WNT5A*, *MYB*, and *FOXM1* mRNAs in one graph. Normal CD4^+^ T cells show low levels of all three, whereas ATL cells show increases in *WNT5A* mRNA levels (indicated by the bubble size) with increasing levels of *MYB* mRNA and *FOXM1* mRNA (Fig. 3B). Then, we examined the relationship between the function of these two transcription factors and the expression level of the *WNT5A* gene by ChIP assays and promoter-reporter assays. First, it was found that c-Myb and FoxM1 were recruited to each other's promoter region and trans-activated each other. Furthermore, ChIP and promoter reporter assays confirmed that both c-Myb and FoxM1 were recruited to the *WNT5A* promoter and promoted transcription (Fig. 3C). Indeed, *WNT5A* promoter activity increased synergistically when c-Myb and FoxM1 were co-expressed compared to c-Myb or FoxM1 alone (Fig. 3D). Also, the effect was further increased by a c-Myb mutant, c-Myb-9A (Fig. 3D).

### Characteristics of the mutant Wnt5a protein overexpressed in ATL cells

**Figure 4:**
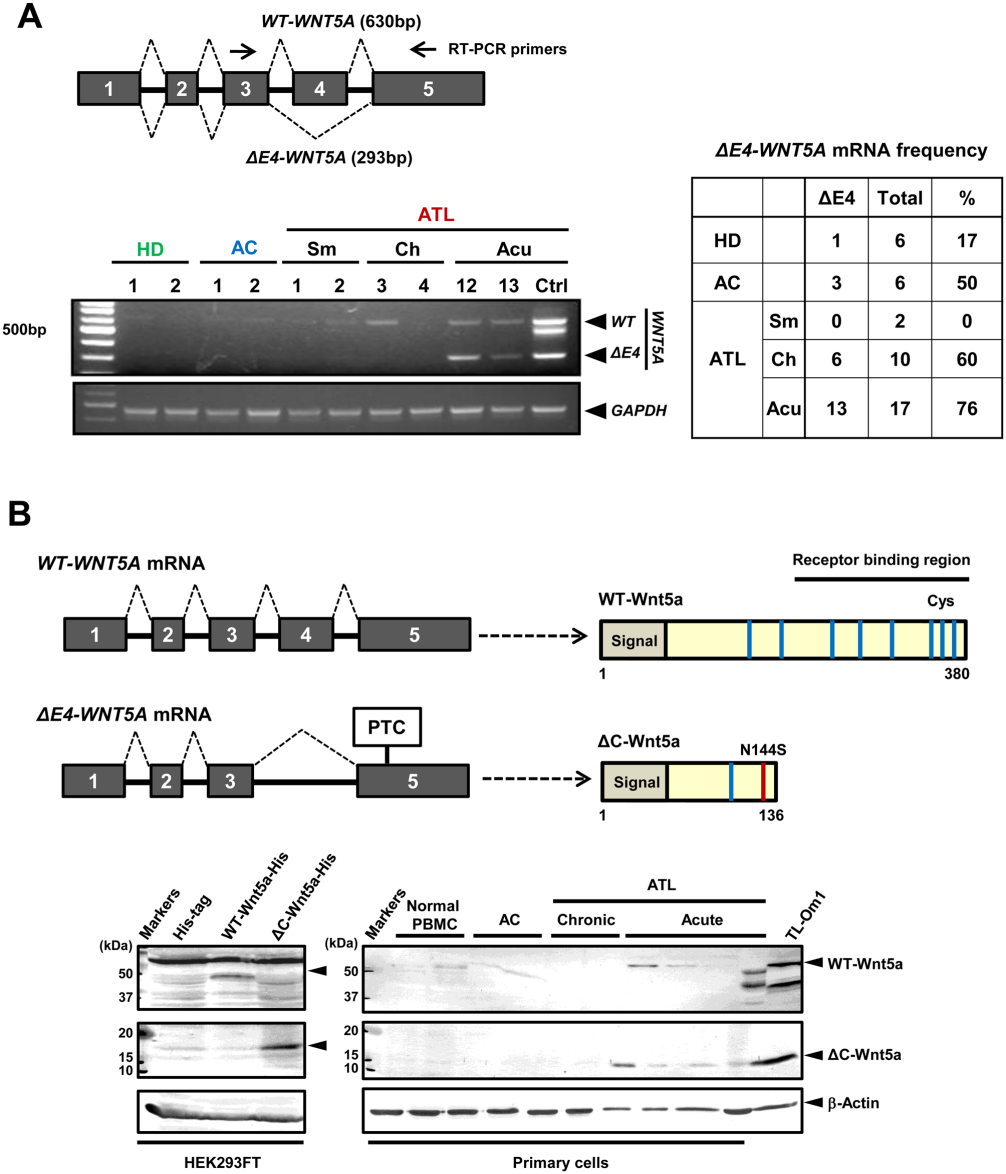
The *ΔExon4-WNT5A* transcript variant and the ΔC-Wnt5a are frequently overexpressed in acute-type ATL cells. (**A**) The semi-quantitative RT-PCR for the *WNT5A* mRNA in total RNA samples extracted from normal CD4^+^ T cells (n = 6), HTLV-1 asymptomatic carriers (ACs; n = 6), and ATL patients (smoldering type; n = 2, chronic type; n = 10, and acute type; n = 17). We designed the primers at the exon3 and exon5 of human *WNT5A* mRNA, which amplifies a 630 bp fragment for the *WT-WNT5A* mRNA, while a 293 bp fragment for the *WNT5A* mRNA without the exon4 (the *ΔE4-WNT5A* mRNA). The left-hand side panel shows the representative data. The rest of the data are shown in Figure S1. Please note that the left-hand side panel consists of two pictures from two separated agarose gels for *WNT5A* mRNA and *GAPDH* mRNA, respectively, which are clearly separated by black frames. Semi-quantitative RT-PCR with these primers showed that the frequency of the *ΔE4-WNT5A* mRNA reached 76% in acute type ATL patients, followed by 60% in chronic type ATL patients (the right-hand side table). (**B**) The *ΔE4-WNT5A* mRNA encodes a C-terminal truncated short Wnt5a isoform of 136 amino acids (the upper panel). The lower panel is the Western blotting with anti-Wnt5a antibody against the N-terminus epitope. The results show the bands of the ΔC-Wnt5a at 15 kDa are detected only in acute-type ATL samples (the right panel). The left panel shows the Western blotting of the WT-Wnt5a-His and the ΔC-Wnt5a-His overexpressed in HEK293FT cells as the reference. Please note that both the left- and the right-hand side panels consist of two pictures from separated blots for Wnt5a and β-Actin, respectively, which are clearly separated by black frames.

Results so far show the drastic overexpression of the *WNT5A* mRNA and its mechanism in ATL cells. However, the structure of *WNT5A* mRNA transcript variant overexpressed in ATL cells has not been clarified. The semi-quantitative RT-PCR of *WNT5A* mRNA in ATL cells showed that a variant *WNT5A* mRNA, which is shorter than the *WT-WNT5A* mRNA, was overexpressed frequently, especially in acute-type ATL cells (Fig. 4A and S1). Sequence analysis of the variant *WNT5A* mRNA revealed the deletion of the fourth exon (Fig. 4A). Thus, the variant is referred to as the *ΔE4-WNT5A* mRNA hereafter. In the *ΔE4-WNT5A* mRNA, the appearance of PTC (premature termination codon) in exon5 causes translation of a mutant Wnt5a that lacks most of the C-terminal region (Fig. 4B, top panel). So, this mutant is called the ΔC-Wnt5a hereafter. In PBMCs from acute-type ATL patients, expression of the ΔC-Wnt5a was confirmed by Western blotting using a Wnt5a antibody, of which epitope is the N-terminal region of Wnt5a. A band was detected at the expected 15 kDa (Fig. 4B lower diagram). The ΔC-Wnt5a band was not clearly detected in PBMCs from healthy donors, asymptomatic HTLV-1 carries (ACs), and chronic-type ATL patients.

### The ΔC-Wnt5a is secreted extracellularly

**Figure 5:**
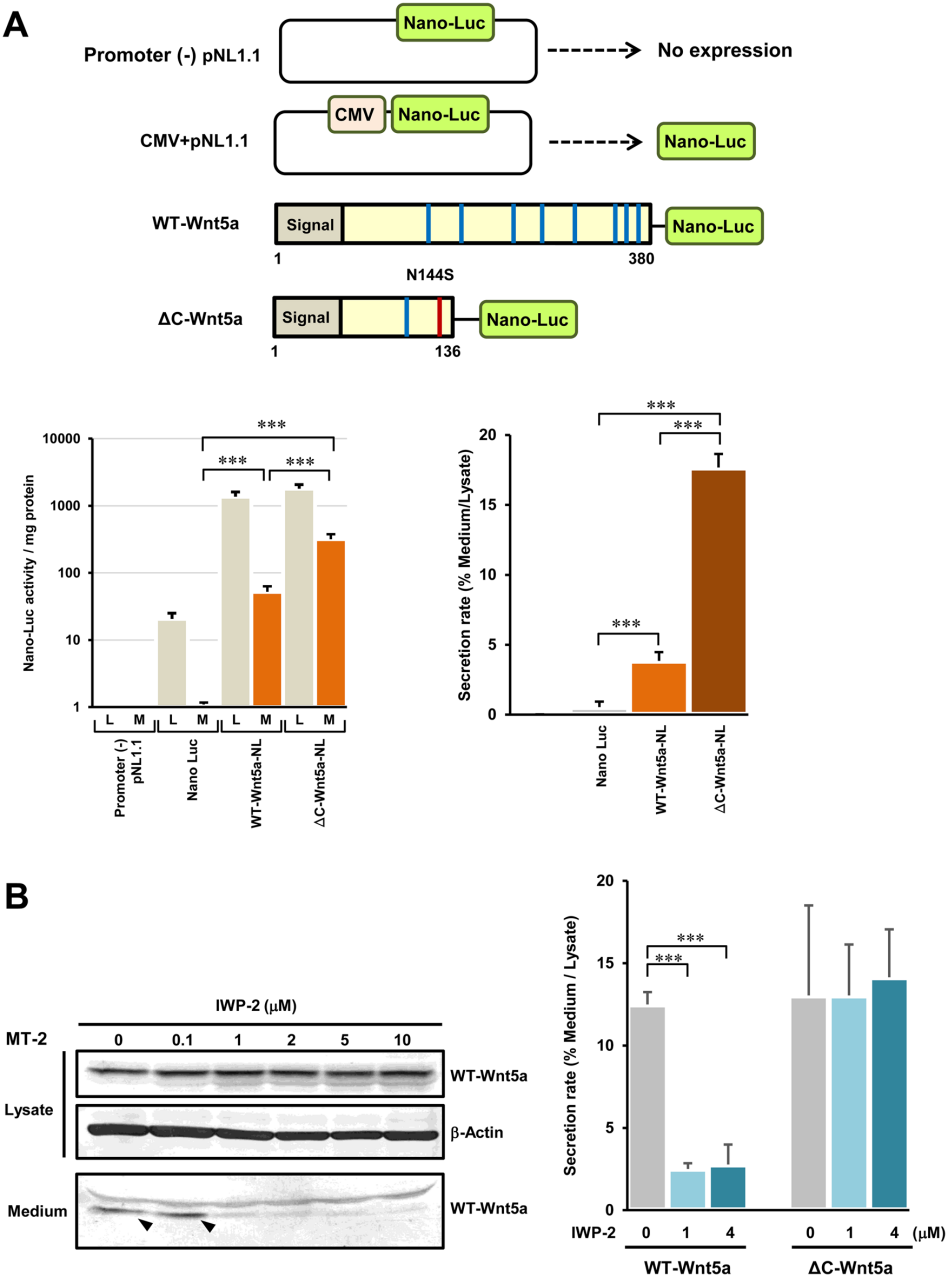
The ΔC-Wnt5a is secreted via a non-canonical secretion pathway. (**A**) In order to test if the ΔC-Wnt5a is secreted out of the cell, we design an assay using Wnt5a fused with NanoLuc at C-terminal (the upper panel). The NanoLuc assays were conducted in the whole cell lysate (L) and the culture medium (M) of HEK293FT cells overexpressing Wnt5a-NanoLuc (the lower left-and side graph, n = 4, mean ± SD, ***P < 0.001), and the secretion rate was calculated as (NanoLuc signal in medium/NanoLuc signal in cell lysate) (the lower right-hand side graph, n = 4, mean ± SD, ***P < 0.001). The secretion rate was about 4-times higher in the ΔC-Wnt5a compared with the WT-Wnt5a. (**B**) IWP-2 inhibits the secretion of Wnt family proteins, including Wnt5a. IWP-2 treatments in MT-2 cells reduced the Wnt5a level detected in the culture medium in a dose-dependent manner, which confirmed that IWP-2 indeed inhibited the secretion of Wnt5a in MT-2 cells (the left-hand side panel). Please note that the left-hand side panel consists of three pictures from three separated blots for the WT-Wnt5a and β-Actin in cell lysate and the WT-Wnt5a in culture medium, respectively, which are clearly separated by black frames. The right-hand side graph shows the secretion rates of the WT-Wnt5a and the ΔC-Wnt5a overexpressed in HEK293FT cells, which were treated with IWP-2 of indicated doses (n = 4, Mean ± SD, ***P < 0.001). The IWP-2 treatment reduced the secretion rate of the WT-Wnt5a significantly, while it did not alter the secretion rate of the ΔC-Wnt5a.

For further investigation of the extracellular secretion characteristics of the ΔC-Wnt5a, the WT- and the ΔC-Wnt5a expression plasmids with NanoLuc (Promega, Corp.) at the C-terminus were prepared (Fig. 5A, upper panel). These plasmids were introduced into HEK293FT cells, and NanoLuc activity in the whole cell lysate (L) and the medium (M) was measured. As results, both the WT-Wnt5a-NanoLuc and the ΔC-Wnt5a-NanoLuc were detected in the cell lysate and the medium, so both types of Wnt5a were confirmed to be secreted extracellularly (Fig. 5A, lower left graph). Furthermore, the secretion rate (NanoLuc activity in the medium/NanoLuc activity in the lysate) was significantly higher in the ΔC-Wnt5a than in the WT-Wnt5a (Fig. 5A, lower right graph).

The ΔC-Wnt5a lacks most of the C-terminal modification site required for normal secretion. Thus, it was predicted to be secreted by a different pathway from the WT-Wnt5a. Therefore, we examined the effect of IWP-2, a Wnt5a secretion inhibitor (Fig. 5B). First, the effect of IWP-2 on the secretion amount of the WT-Wnt5a was examined, using HTLV-1-infected immortalized cell line MT-2. As a result, the amount of the WT-Wnt5a in the medium decreased depending on the concentration of IWP-2. From this result, it was confirmed that IWP-2 inhibits the secretion of the WT-Wnt5a via the Golgi apparatus and secretory vesicles (Fig. 5B, left panel). Next, we treated HEK293FT cells expressing the WT-Wnt5a-NanoLuc or the ΔC-Wnt5a-NanoLuc with IWP-2 of various concentrations, and measured NanoLuc activity in the medium. As a result, the amount of the WT-Wnt5a-NanoLuc in the medium was significantly reduced by treatment with IWP-2 of 1 μM or more, but the amount of the ΔC-Wnt5a-Nano-Luc did not change with 4 μM IWP-2 (Fig. 5B, right graph). These results suggest that the ΔC-Wnt5a is secreted through a pathway different from the WT-Wnt5a secretion pathway.

### Effect of the ΔC-Wnt5a on cell motility

**Figure 6:**
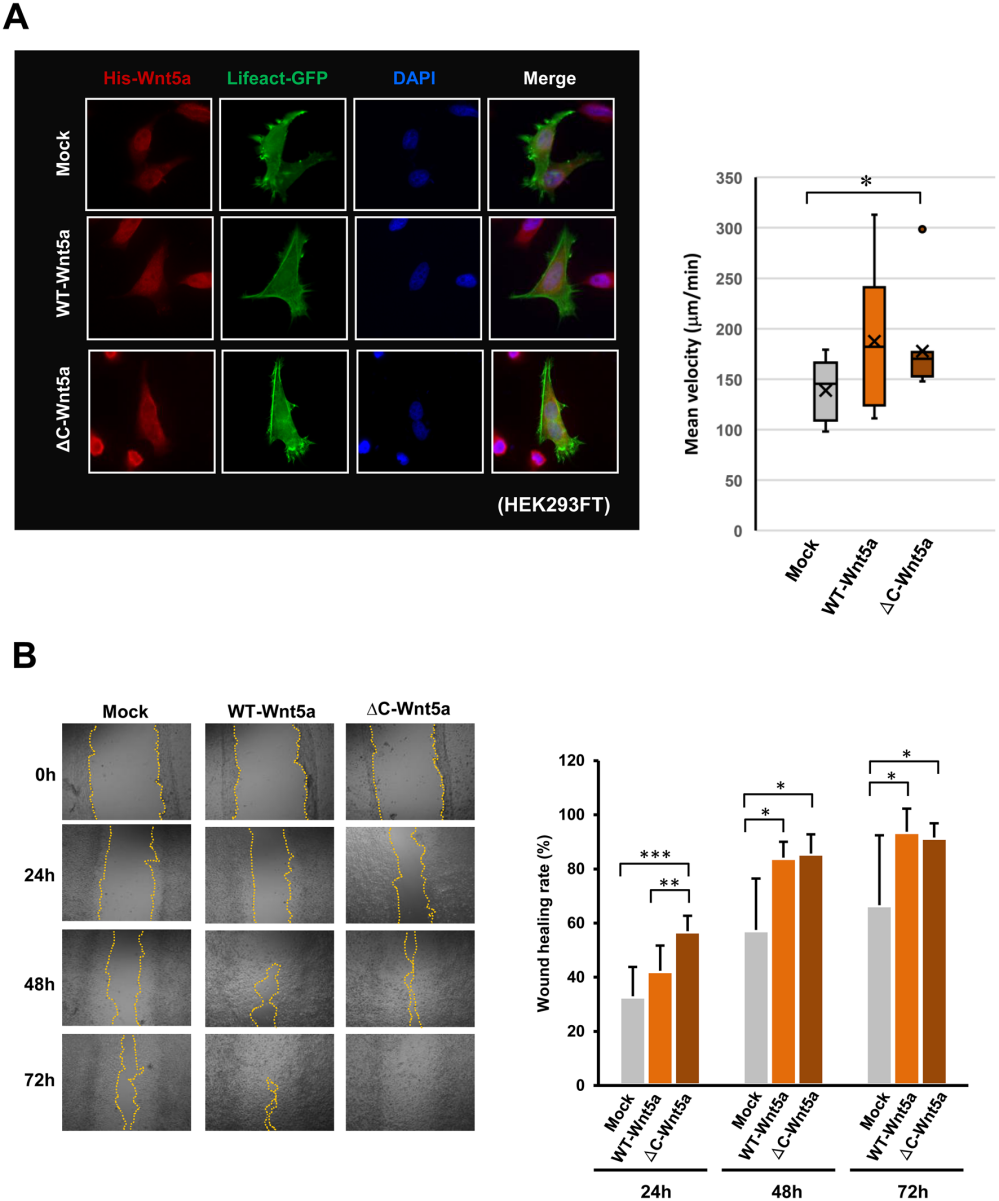
The ΔC-Wnt5a induces a higher cell velocity and an invasion rate than the WT-Wnt5a. (**A**) HEK293FT cells overexpressing Wnt5a-His and Lifeact-GFP were subjected to the cell velocity analysis. The movements of HEK293FT cells (expressing mock, the WT-Wnt5a-His, or the ΔC-Wnt5a-His, 10 cells each) were monitored with BioStation live cell imaging system (Nikon, Corp.), and the mean velocity was calculated using ADAPT software. The analysis shows that the mean velocity significantly increased in the ΔC-Wnt5a overexpressing cells compared with the mock cells with the empty vector (n = 10, mean ± SD, *P < 0.05). (**B**) Wound-healing assays were conducted in HEK293FT cells overexpressing the empty vector (mock), the WT-Wnt5a-His, or the ΔC-Wnt5a-His. The wound-healing rate was calculated at 24, 48, and 72 h after the wound formation as {100% − [(wound area at the time)/(wound area at 0 h) × 100%)]}. The graph demonstrates that Wnt5a expressing cells show significantly higher wound-healing rates compared with the mock control cells. Moreover, the wound-healing rate of the ΔC-Wnt5a expressing cells was significantly higher than the WT-Wnt5a expressing cells at 24 h (n = 6, mean ± SD, *P < 0.05; **P < 0.01; ***P < 0.001).

The results so far indicate that the ΔC-Wnt5a is secreted extracellularly and may act on surrounding cells. Therefore, we evaluate the effect of this mutant Wnt5a on cell motility. Using HEK293FT cells overexpressing the WT-Wnt5a-His or the ΔC-Wnt5a-His, the effect on cell migration rate was examined. The Lifeact-GFP that binds explicitly to polymerized actin was simultaneously expressed, and the morphology of the cells was also observed (Fig. 6A, left panel). The mean velocity was calculated using ADAPT software based on 10 time-lapse images of the mock, the WT-Wnt5a, and the ΔC-Wnt5a expressing cells (n = 10). As a result, the cell migration rate was significantly increased in cells expressing the ΔC-Wnt5a compared to the mock cells (Fig. 6A, right graph). The wound-healing assay in HEK293FT cells expressing the WT-Wnt5a-His or the ΔC-Wnt5a-His showed that the wound healing rate in the cells expressing the ΔC-Wnt5a was the fastest. Especially at 24 h, it was significantly higher than WT-Wnt5a-expressing cells (Fig. 6B).

### Effect of the ΔC-Wnt5a on T-cell invasion activity

**Figure 7:**
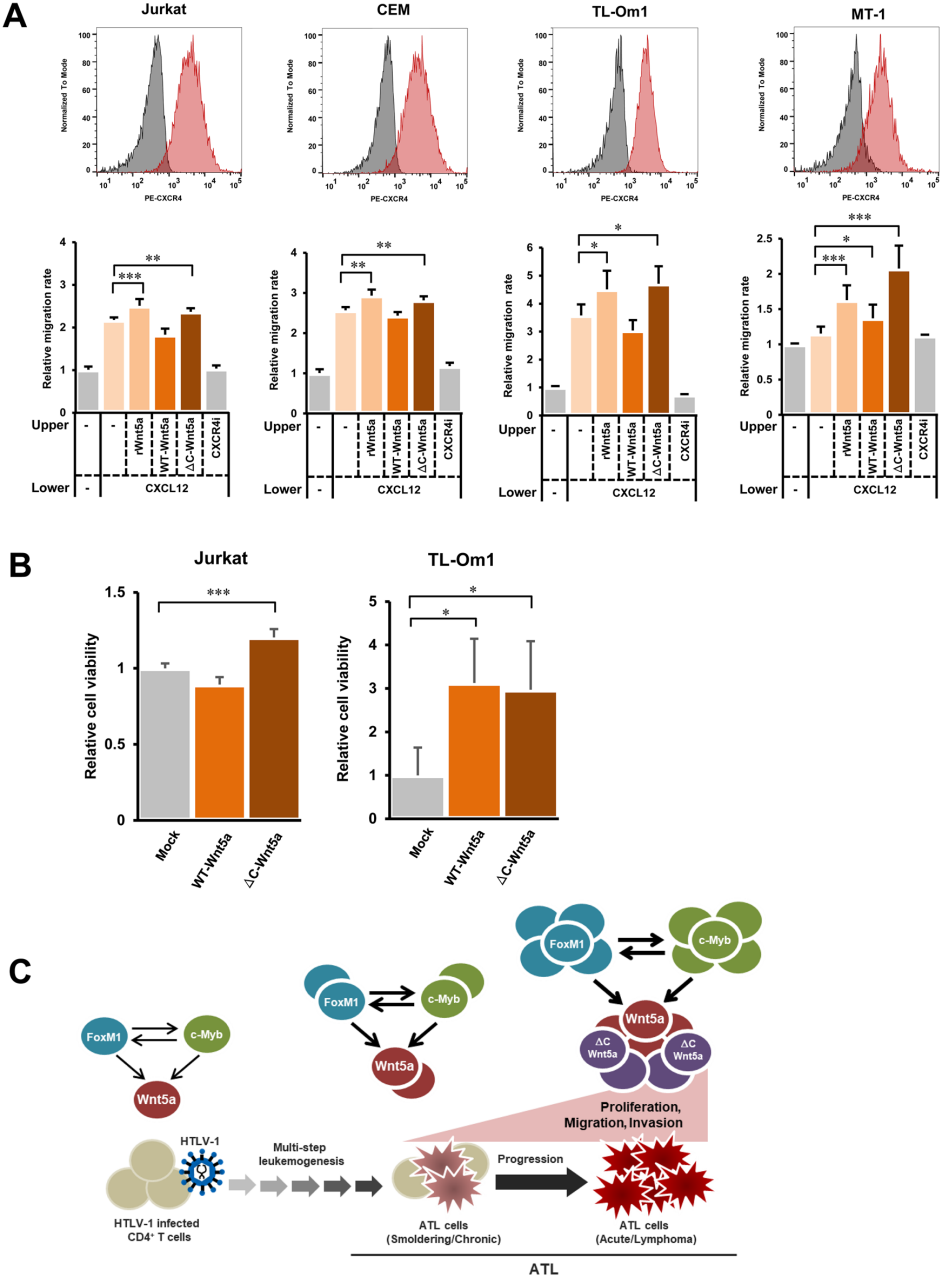
The ΔC-Wnt5a induces strong chemotaxis to CXCL12 and support cell growth. (**A**) The upper panels show the expression of CXCR4 in Jurkat, CEM, TL-Om1, and MT-1 cells measured by flowcytometry. The lower graphs show the relative migration rates toward CXCL12 in Jurkat, CEM, TL-Om1, and MT-1 cells incubated in the medium containing the WT-Wnt5a or the ΔC-Wnt5a (n = 6, mean ± SD, *P < 0.05; **P < 0.01; ***P < 0.001). Recombinant human Wnt5a (rWnt5a) was added at 100 ng/mL to the medium as a positive control, which induced significantly higher migration rates compared with the negative controls (medium only) in all tested cell lines. The WT-Wnt5a did not stimulate the cellular chemotaxis to CXCL12 except for MT-1. The ΔC-Wnt5a significantly enhanced the chemotaxis to CXCL12 in all cell lines to the same extent compared with the rWnt5a. The treatment with a CXCR4 antagonist (CXCR4i) abolished the cellular migration to CXCL12 in all cell lines. Thus, CXCL12/CXCR4 chemotaxis played a major role in the cellular migration observed in these assays. (**B**) The cell viability assays were conducted in Jurkat and TL-Om1 cells cultured in the medium containing the WT-Wnt5a or the ΔC-Wnt5a. The relative viability was significantly elevated in the both cell lines, which were cultured in the medium containing the ΔC-Wnt5a compared with the cells cultured in mock medium (n = 6, mean ± SD, *P < 0.05; ***P < 0.001). The relative cell viability was significantly elevated only in TL-Om1 cells, which were cultured in the medium containing the WT-Wnt5a (n = 6, mean ± SD, *P < 0.05). (**C**) Summary of this study. We demonstrated that both FoxM1 and c-Myb transactivate Wnt5a expression. Since FoxM1 and c-Myb transactivate each other, elevated levels of FoxM1 and c-Myb in ATL cells synergistically enhance the Wnt5a expression. Such feed-forward molecular mechanism may cause the drastic overexpression of Wnt5a in acute-type ATL cells. Moreover, in acute-type ATL cells, the Wnt5a level is not only elevated, but also a mutant, yet functional Wnt5a, the ΔC-Wnt5a, is overexpressed. Overexpression of the ΔC-Wnt5a may accelerate the dysregulation in the Wnt5a pathway, which may be responsible for malignant phenotypes of acute-type ATL cells, such as uncontrollable cell proliferation, migration, and invasion.

Chemotaxis via the CXCR4/CXCL12 axis is essential for T-cell migration. Indeed, CXCR4 is overexpressed in T-cell lines, including HTLV-1 related T-cell lines, and in primary ATL cells (Fig. 7A and Fig. S2). Thus, we examined the effects of the ΔC-Wnt5a on the CXCR4/CXCL12-dependent chemotaxis of T cells. The culture medium of HEK293FT cells overexpressing the WT-Wnt5a or the ΔC-Wnt5a was added to the medium of T-cell lines (Jurkat, CEM, TL-Om1, and MT-1) and chemotaxis toward CXCL12 was evaluated. Human recombinant Wnt5a (rWnt5a, 100 ng/mL) was used as a positive control. As shown in Fig. 7A, the addition of rWnt5a significantly increased the migration rate for CXCL12 in all T-cell lines. On the other hand, the CXCR4 antagonist (CXCR4i) canceled the migration induced by rWnt5a. Thus, it was confirmed that the CXCR4/CXCL12-dependent migration caused by Wnt5a was evaluated in this experiment. In this experimental system, the ΔC-Wnt5a significantly increased the migration rate toward CXCL12 in any tested T-cell line and showed a tendency to be higher than the WT-Wnt5a. Therefore, the ΔC-Wnt5a is expected to enhance the CXCR4/CXCL12-dependent T-cell migration rate compared to the WT-Wnt5a.

### Effects of the ΔC-Wnt5a on cell viability

Finally, we examined if the ΔC-Wnt5a affects the cell viability. Figure 7B demonstrated that the ΔC-Wnt5a significantly enhanced the cell viability in both Jurkat and TL-Om1 cells, while the WT-Wnt5a enhanced the cell viability only in TL-Om1. To elucidate how the ΔC-Wnt5a enhances cell viability, we investigated the effects of Wnt5a on activation of the NF-κB and c-Src pathways. As a result, NF-κB was significantly activated in HEK293FT cells overexpressing the ΔC-Wnt5a (Fig. S3A). Moreover, the activated c-Src, which is phosphorylated at Y416, P-Src (Tyr416), was detected at a high-level in the ΔC-Wnt5a-expressing cells (Fig. S3B). These results indicate that the ΔC-Wnt5a promotes the activation of c-Src, thus activates the NF-κB pathway to maintain a high cell viability. Indeed, we confirmed the recovery of cell growth in Wnt5a-knockdown cells when the ΔC-Wnt5a was conditionally knocked-in by using a doxycycline-dependent Tet-on system (Fig. S4).

## Discussion

In the present study, we revealed that the total *WNT5A* mRNA expression level in acute-type ATL cells is more than 100-folds higher than indolent-type ATL cells (Fig. 1C). Overexpression of *WNT5A* mRNA in HTLV-1 infected cell lines and PBMCs derived from ATL patients (Fig. 1A,B) has already been reported^23,24^. However, PBMCs include B cells, natural killer cells, monocytes, granulocytes, and dendritic cells in addition to T cells. To understand abnormalities specific to ATL cells (leukemic T cells), we focused on the tumor cell fraction (CADM1+/CD7−) enriched by the HAS-Flow method^26^. Our results suggest that overexpression of Wnt5a may be closely related to the high organ infiltration and metastatic potential of acute ATL cells associated with ATL progression. Indeed, Wnt5a-dependent cell viability was observed in MT-2 and TL-Om1 cells with high endogenous Wnt5a expression levels (Fig. 2A,C). Inhibition of Wnt5a by IWP-2 treatment also reduced NF-κB activity (Fig. 2B). It has been reported that Wnt5a activates the NF-κB pathway^10,27^. Thus, overexpression of Wnt5a may be responsible for the cell proliferation via NF-κB activation.

Next, we examined upstream regulators of Wnt5a expression in ATL cells (Fig. 3). Wnt5a is known to be overexpressed in many cancers, but the molecular mechanism of its overexpression is poorly understood. It has been reported that Hbz, which is encoded by the antisense HTLV-1 genome, is involved in the regulation of the Wnt5a overexpression in ATL cells^24^. In the present study, we focused on the overexpression of two proto-oncogenic transcription factors, FoxM1 and c-Myb, as upstream regulators of the *WNT5A* gene expression in ATL cells. FoxM1 is a transcription factor, which belongs to the Forkhead box family. FoxM1 is a critical regulator in cell-cycle, cell differentiation, and DNA repair. Especially, FoxM1 is involved in DNA-damage repair response and promotes G2/M progression by transactivating vital cell-cycle regulator genes, such as *CKNA*, *CKNB*, *CDC25B*, *AURKA/B*, and *PLK1*^28,29^. Overexpression of FoxM1 has been reported in various carcinomas and lymphomas/leukemias^30–33^. C-Myb is a proto-oncoprotein, which regulates the development and proliferation of hematopoietic cells as a transcription factor^34–37^. Overexpression and deregulation of c-Myb functions have been also frequently observed in hematopoietic tumors, especially in AML, ALL, and lymphomas, and the implications of c-Myb overexpression/deregulation and leukemogenesis have been extensively investigated^38–41^. Recently, the authors reported the overexpression of c-Myb in ATL cells^42^. Importantly, FoxM1 has been reported as a transcription factor of the *WNT5A* gene^33^. Also, c-Myb was reported to function as a feed-forward activating partner of FoxM1^43^. Therefore, we speculated that the overexpression of c-Myb would enhance the FoxM1 expression, thus the Wnt5a expression. Our gene expression analysis confirmed correlated increases of *FOXM1*, *MYB*, and *WNT5A* mRNAs in ATL cells compared with normal CD4^+^ T cells (Fig. 3A,B). Correlation among those three mRNAs let us investigate the molecular relationship of FoxM1 and c-Myb in Wnt5a overexpression. Firstly, we confirmed that c-Myb and FoxM1 transactivate each other. Then we also demonstrated that the *WNT5A* gene was transactivated by FoxM1 and c-Myb (Fig. 3C). In particular, it was shown that the c-Myb-9A mutant overexpressed in ATL cells^42^ significantly increased *WNT5A* promoter activity compared to the WT-c-Myb (Fig. 3C). Finally, we demonstrated that the activity of the *WNT5A* promoter increased synergistically under co-expression of c-Myb and FoxM1 as compared to the expression alone (Fig. 3D). Moreover, when FoxM1 and c-Myb-9A were co-expressed, the *WNT5A* promoter activity was increased about sixfold as compared to FoxM1 alone, and more than twofold as compared to the co-expression of FoxM1 and WT-c-Myb (Fig. 3D). Based on these results, we speculate that overexpression of FoxM1 and c-Myb amplify molecular dysfunction by activating each other's transcription, thus synergistically activate transcription of common target genes, including the *WNT5A* gene.

In the present study, we demonstrated not only overexpression of the total *WNT5A* gene, but also selective elevation of a variant *WNT5A* mRNA, the *ΔE4-WNT5A* mRNA (Fig. 4A), and its product mutant Wnt5a, the ΔC-Wnt5a (Fig. 4B), in acute-type ATL cells. Hence, our next question was if the ΔC-Wnt5a is biologically functional. The secretion and function of Wnt5a are finely regulated by posttranslational lipid modifications^44^. The ΔC-Wnt5a lacks the C-terminal lipid modification sites, as well as sugar-chain modification sites, both of which are thought to be involved in extracellular secretion and function of Wnt5a. Surprisingly, the ΔC-Wnt5a showed a significantly higher secretion rate compared to the WT-Wnt5a (Fig. 5A). We speculate that the ΔC-Wnt5a is released extracellularly via a pathway different from the canonical secretion pathway of the WT-Wnt5a, because the secretion of the ΔC-Wnt5a was not stopped by IWP-2, which inhibits lipid modification essential for Wnt secretion (Fig. 5B). Upon confirming that the ΔC-Wnt5a was released extracellularly, we examined the function of the ΔC-Wnt5a next. Wnt5a binds to receptors such as Frizzled and promotes actin polymerization, thus cell motility^7,13,14^. The migration rate of HEK293FT cells overexpressing the ΔC-Wnt5a was higher than that of the WT-Wnt5a-expressing cells (Fig. 6). We speculate that ectopically expressed Wnt5a proteins were secreted and stimulated the cell migration in an autocrine manner. It has been reported that Wnt5a enhances the chemotaxis of cells via CXCR4/CXCL12^45^. To further investigate the underlying molecular mechanism of the cell motility enhanced by Wnt5a, we performed a migration/invasion assay toward CXCL12 in the T-cell lines cultured in the WT-Wnt5a or the ΔC-Wnt5a containing medium. As a result, the ΔC-Wnt5a significantly increased cell invasion activity toward CXCL12, while the WT-Wnt5a did not, in all tested cell lines expressing high levels of CXCR4 (Fig. 7A). Since CXCR4 is also overexpressed in primary acute-type ATL cells (Fig. S2), we speculate that CXCR4/CXCL12 dependent cell migration may be also enhanced by the ΔC-Wnt5a in primary ATL cells in an autocrine manner.

Finally, we investigated the function of the ΔC-Wnt5a in cell viability (Fig. 7). The ΔC-Wnt5a significantly enhanced the cell viability in Jurkat and TL-Om1 (Fig. 7B). Unexpectedly, the WT-Wnt5a also significantly elevated the cell viability of TL-Om1 cells. Unlike Jurkat cells, TL-Om1 cells express the ΔC-Wnt5a endogenously (Fig. 4B). Therefore, the effects of the ectopically administrated WT-Wnt5a on the cell viability may have been influenced by the endogenous ΔC-Wnt5a in TL-Om1 cells. Since the conditional knock-in of the ΔC-Wnt5a restored the cell-growth rate in the *WNT5A* knockdown TL-Om1 cells (Fig. S4), we speculate that the ΔC-Wnt5a also plays the central role in enhancement of cell viability in TL-Om1 cells. It has been reported that Wnt5a activates the NF-κB pathway and the Src pathway, thus contributes to the maintenance of cell proliferation^22^. Consequently, we examined the effects the ΔC-Wnt5a on NF-κB activity and induction of activated P-Src (Tyr416). The results showed that the ΔC-Wnt5a activated the NF-κB pathway (Fig. S3A) and the c-Src pathway (Fig. S3B) more significantly than the WT-Wnt5a. These results suggest that the ΔC-Wnt5a enhances cell viability via activation of the NF-κB and the Src pathways.

Nevertheless, how the ΔC-Wnt5a without essential glycosylation sites maintains its function is required to be elucidated in the future. The activity of the Wnt pathway is regulated by inhibitors such as WIF1 (Wnt inhibitory factor 1) and SFRP1 (Secreted frizzled-related protein 1) that inhibit receptor-binding of Wnt proteins^46^. To better understand the function of the ΔC-Wnt5a, it is also necessary to examine the interaction between the ΔC-Wnt5a and those Wnt inhibitors in the future.

Taken together, we demonstrate that the total *WNT5A* gene expression is elevated 100-fold in acute-type ATL cell compared with indolent-type ATL cells. Also, we propose a high possibility that feed-forward synergistic activations of FoxM1 and c-Myb are responsible for such drastic increase of the *WNT5A* gene expression. Moreover, a variant *WNT5A* mRNA lacking the exon4 (the *ΔE4-WNT5A* mRNA) was overexpressed in acute-type ATL cells. The ΔC-Wnt5a encoded by the *ΔE4-WNT5A* mRNA maintains biological function of Wnt5a in higher magnitude than the WT-Wnt5a. Consequently, these results support the notion that the ΔC-Wnt5a overexpression may be deeply related to the malignant phenotypes of the acute-type ATL cells, such as uncontrollable proliferation and metastatic invasion, via amplifying the disorders in the Wnt5a pathway (Fig. 7C).

## Methods

Experimental procedures in the current study are explained below. Please note that we follow appropriate guidelines, methods reported elsewhere or provided by manufactures to conduct experiments in this study.

### Cell lines and cell cultures

All cell lines, Jurkat, CEM, Molt4 (T-ALL patient-derived T-cell lines), TL-Om1, MT-1, HUT102 (ATL patient-derived T-cell lines), MT-2, and C91/PL (HTLV-1-immortalized T-cell lines), used in the present study were obtained and maintained, as previously reported^42^ and as explained in the Supplementary Information S1. Also, we authenticated those cell lines within 6 months of experiments as explained in the Supplementary Information S1. Especially, we confirmed by fluorescent in situ hybridization and HTLV-1 provirus-specific real-time PCR that TL-Om1 contained one copy/cell of HTLV-1 provirus at the site of 1p13 of chromosome 1 in the genomic DNA, maintaining the original characteristics of TL-Om1^47^. Also, we authenticated MT-2 within six months of this study by the proviral integration-site sequencing technique^48^ and confirmed that this cell line contains ten copies of HTLV-1 provirus/cell.

### Preparation of primary cells

Peripheral blood mononuclear cells (PBMCs) from ATL patients, HTLV-1 asymptomatic carriers, and healthy donors were isolated for experiments as shown in the Supplementary Information S1. The clinical information of ATL patients, HTLV-1 asymptomatic carriers, and healthy donors enrolled in the present study is also shown in Supplementary Table S1. The primary samples used in the present study were a part of those collected with informed consent as a collaborative project of the Joint Study on Prognostic Factors of ATL Development (JSPFAD). The project was approved by the Human Genome Research Ethics Committee in the Institute of Medical Sciences, the University of Tokyo (IMSUT).

### Construction of protein expression plasmids

Mammalian cell expression plasmids of WT-Wnt5a and ΔC-Wnt5a with His-tag or NanoLuc at the C-terminus were constructed in the present study. Also, His-FoxM1, FLAG-WT-c-Myb, and FLAG-c-Myb-9A expression plasmids were prepared for ChIP assays. The FoxM1, WT-c-Myb, and c-Myb-9A without tags were used in the promoter-luciferase reporter assay. The detailed methods and primers used in constructions of those plasmids are available in the Supplementary Information S1.

### ChIP (chromatin immunoprecipitation) assays against c-Myb and FoxM1

To analyze the recruitment of c-Myb on *FOXM1* gene and *WNT5A* gene, as well as that of FoxM1 on *MYB* gene and *WNT5A* gene, we conducted ChIP assays against c-Myb and FoxM1 in HEK293FT cells, which transiently overexpressed FLAG-c-Myb or His-FoxM1. We employed ChIP procedure described previously^42^ with some modifications. More detailed ChIP procedure is shown in the Supplementary Information S1.

### Firefly-luciferase based promoter activity assays

To evaluate the activation of *FOXM1* promoter and *WNT5A* promoter by c-Myb, as well as that of *MYB* promoter and *WNT5A* promoter by FoxM1, firefly-luciferase based promoter reporter assays were conducted. Briefly, the promoter-reporter plasmid and effector plasmid(s) (i.e., WT-c-Myb-pCDNA3, c-Myb-9A-pCDNA3 and/or FoxM1-pCDNA6B) were transfected to HEK293FT cells by PEI. After 24 h, the firefly-luciferase activity was measured by the dual luciferase assay system (Promega, Corp.). The detailed method and primers used for constructions of promoter-reporter plasmids are described in the Supplementary Information S1.

### Firefly-luciferase based NF-κB activity reporter assay

In order to examine the effect of Wnt5a inhibition on the cellular NF-κB activity,

Firefly-luciferase based NF-κB activity reporter assays were conducted in Jurkat and TL-Om1 cells under IWP-2 treatments. Also, effects of the WT-Wnt5a and the ΔC-Wnt5a on the cellular NF-κB activity were analyzed in HEK293FT cells transfected with a NF-κB activity reporter plasmid together with WT-Wnt5a-pCDNA6B or ΔC-Wnt5a-pCDNA6B. Please see the Supplementary Information S1 for more detail.

### The Wnt5a secretion assay

The level of secreted Wnt5a was evaluated by the signal of NanoLuc (Promega, Corp.). NanoLuc is an artificially designed luciferase of 19 kDa, which is smaller than classical firefly- or *Renilla*-luciferases (62 kDa and 36 kDa, respectively). Because of smaller molecular weight, NanoLuc does not hinder the secretion of recombinant proteins with signal peptides for extra-cellular secretion. Therefore, we designed the expression plasmids of the WT-Wnt5a and the ΔC-Wnt5a infusion with NanoLuc at the C-terminal. The WT-Wnt5a -NanoLuc or the ΔC-Wnt5a-NanoLuc was overexpressed in HEK293FT cells for 24 h, and NanoLuc activity was measured in the culture medium and the whole-cell lysate, separately, using NanoLuc Luciferase Technology (Promega, Corp.). The secretion rate was calculated as % (NanoLuc activity in the medium)/(NanoLuc activity in the cells). Construction of the WT-Wnt5a-NanoLuc and the ΔC-Wnt5a-NanoLuc expression plasmids are shown in the Supplementary information S1.

### Measurement of mean cellular velocity

In order to clarify autocrine/paracrine effects of secreted Wnt5a on the cellular motility, we measured the mean velocity of the HEK293FT cells overexpressing the WT-Wnt5a or the ΔC-Wnt5a. We observed the movement of the cells by live-cell imaging with BioStation IM (Nikon, Corp.) and calculated the mean velocity of each cell using ADAPT: Automated Detection and Analysis of ProTrusions^49^. More detailed methods are found in the Supplementary Information S1.

### Wound-healing assay

To further evaluate the effects of the WT-WNt5a and the ΔC-Wnt5a on the cell motility, we employed the wound-healing assay in HEK293FT cells overexpressing the WT-WNt5a or the ΔC-Wnt5a. The detailed method of the assay is described in the Supplementary Information S1.

### CXCR4 expression levels

Prior to CXCR4/CXCL12 dependent chemotaxis assay, we examined the basal CXCR4 expression levels in Jurkat, CEM, TL-Om1, MT-1, MT-2, and PBMC from a chronic-type ATL patient by flow-cytometry. Then, we determined to use Jurkat, CEM, TL-Om1, and MT-1 in the following chemotaxis assay.

### The CXCR4/CXCL12-dependent chemotaxis assay

Stimulation of CXCR4/CXCL12 dependent chemotaxis by WT-Wnt5a and ΔC-Wnt5a was examined by chemotaxis assay. In this assay, Jurkat, CEM, MT-2, and TL-Om1 cells were cultured in the WT-Wnt5a or the ΔC-Wnt5a containing HEK293FT culture medium in the top chamber and CXCL12-containing RPMI was applied to the bottom chamber of a transwell chemotaxis assay system, The detailed method of the chemotaxis assay is described in the Supplementary information S1.

### The Wnt5a specific knockdown

For specific knockdown of endogenous Wnt5a, two lentiviral plasmids encoding shRNAs specific for Wnt5a were prepared according to the protocol (http://urx3.nu/qzUb) published by Dr. Hiroyuki Miyoshi, RIKEN, Japan. The detailed methods for construction of the lentiviral plasmids, as well as for preparation of shRNA expressing lentivirus are explained in the Supplementary Information S1.

### Conditional knock-in of Wnt5a by a Tet-on system

To examine the isoform-specific effects of the WT-Wnt5a or the ΔC-Wnt5a on cell viability, we employed a Wnt5a Tet-on system. Briefly, we prepared TL-Om1 cells with the *WT-WNT5A* or the *ΔE4-WNT5A* gene regulated by doxycycline-dependent promoter (TL-Om1-Tet-on-WT or TL-Om1-Tet-on-ΔC). Next, we knockdown endogenous Wnt5a expression by lentiviral shRNA in TL-Om1-Tet-on-WT or TL-Om1-Tet-on-ΔC. Then, the expression of the WT-Wnt5a or the ΔC-Wnt5a was induced by doxycycline treatment. The viability of Wnt5a-knockdown cells, i.e., Venus ( +) cells, was examined by flow-cytometry before and after the Tet-on of Wnt5a expression. Preparation of the Wnt5a Tet-on system and the course of the viability assay are explained in the Supplementary Information S1.

### Effect of Wnt inhibitor (IWP-2) on cell proliferation

To analyze the effect of Wnt inhibition on the cell proliferation rate, cell lines (Jurkat, CEM, Molt4, MT-2, HUT102, TL-Om1) were treated with IWP-2, a pan-Wnt protein inhibitor, followed by the cell viability assay. The detailed methods of the IWP-2 treatment and the cell viability assay are shown in the Supplementary Information S1.

### Semi-quantitative RT-PCR and quantitative RT-PCR (qPCR)

In the present study, we evaluated the expression pattern of *WNT5A* splicing variants by the semi-quantitative RT-PCR with the primers, which were designed in the exon3 and the exon5 of the *WNT5A* mRNA. Also, we measured the expression level of total *WNT5A* mRNA in cell lines and primary ATL cells by quantitative RT-PCR (q-PCR) with the primers, which can detect all *WNT5A* variant mRNAs. Preparation of total RNA samples and methods for RT-PCR and q-PCR, as well as the primer sequences used for the PCRs can be found in the Supplementary Information S1.

### Western blotting

We analyzed the endogenous and exogenous Wnt5a protein levels in the cell lysate of cell lines and primary cells by Western blotting with antibodies against Wnt5a or the His-tag. We also examined Src protein levels in HEK293FT cells overexpressing the WT-Wnt5a or the ΔC-Wnt5a. The method to prepare the whole cell lysate and the list of primary and secondary antibodies used in the Western blotting analyses are shown in the Supplementary Information S1.

Images of membranes were scanned by a scanner (#GT-X970, Seiko-Epson Corp.), and digital images were processed using the Photoshop software (Adobe Ltd.). Processing was conducted appropriately only in changing brightness and contrast across the entire image including control and experimental samples. All original images of Western blotting data in the main figures are shown in the Supplementary Information S1.

### Statistical analysis

The statistical difference between experimental groups was evaluated by Student *T*-test throughout this study. Statistical significance is indicated by astariscs (*p < 0.05, **p < 0.01, and ***p < 0.001). The number of the samples (n) in each experimental group is indicated in the figure legend.

## Supplementary Information

Supplementary Information
