# Supplementary material for: Overexpression of aberrant Wnt5a and its effect on acquisition of malignant phenotypes in adult T-cell leukemia/lymphoma (ATL) cells
Source: Sci Rep. 2021 Feb 18;11:4114. doi: 10.1038/s41598-021-83613-2 (PMC7892546; doi:10.1038/s41598-021-83613-2)
Supplement: Supplementary file 1 — Supplementary Information [file 41598_2021_83613_MOESM1_ESM.docx]

**Supplementary information**

**Overexpression of aberrant Wnt5a and its effect on acquisition of malignant phenotypes in adult T-cell leukemia/lymphoma (ATL) cells**

Kazumi Nakano^1^*, Yohei Chihara^1^, Seiichiro Kobayashi^2^, Masako Iwanaga^3^, Atae Utsunomiya^4^, Toshiki Watanabe^5^, and Kaoru Uchimaru^1^

^1^Department of Computational Biology and Medical Sciences, Graduate School of Frontier Sciences, The University of Tokyo, Tokyo, Japan

^2^Department of Hematology, Kanto Rosai Hospital, Kanagawa, Japan

^3^Department of Clinical Epidemiology, Graduate School of Biomedical Science, Nagasaki University, Nagasaki, Japan

^4^Department of Hematology, Imamura General Hospital, Kagoshima, Japan

^5^Laboratory of Practical Management of Medical Information, Graduate School of Medicine, St. Marianna University, Kawasaki, Japan

**Supplementary Methods**

**Cell lines and cell cultures**

Jurkat, CEM, Molt4 (T-ALL patient-derived T-cell lines), TL-Om1, MT-1, HUT102 (ATL patient-derived T-cell lines), MT-2, and C91/PL (HTLV-1-immortalized T-cell lines) were maintained in RPMI (Gibco, Thermo Fisher Scientific, Inc.) containing 10% FBS (Gibco, Thermo Fisher Scientific, Inc) at 37 ℃ with 5% CO_2_. HeLa (cervical cancer-derived epithelial cell line)**,** HEK293T (human embryonic kidney-derived cell line containing the SV40 T-antigen), and HEK293FT (a derivative of HEK293T) cells were cultured in DMEM (Nissui Pharmaceutical Co., Ltd.) containing 10% FBS (Gibco, Thermo Fisher Scientific, Inc.) at 37 ℃ with 5 % CO_2_.

The cell lines used in this study were obtained and authenticated as follows. TL-Om1, derived from malignant T cells from an ATL patient, was kindly provided by Dr. K. Sugamura at Tohoku University, Japan. We authenticated within six months of this study by fluorescent *in situ* hybridization and HTLV-1 provirus-specific real-time PCR. We confirmed that this cell line contained one copy/cell of HTLV-1 provirus at the site of 1p13 of chromosome 1 in the genomic DNA, maintaining the original characteristics of TL-Om1^47^. C91/PL is a kind gift from Dr. Maria-Isabel Thoulouze in the Institute of Pasteur, France, who authenticated and confirmed that this cell line contains one HTLV-1 full-length provirus/cell and produces infectious HTLV-1 virions within six months of this study. MT-2, HTLV-1 transformed T-cell line, was kindly provided by Dr. H. Hoshino in Gunma University, Japan. We authenticated within six months of this study by the proviral integration-site sequencing technique^48^ and confirmed that this cell line contains ten copies of HTLV-1 provirus/cell. We also confirmed that this cell line produced infectious HTLV-1 viral particles and expressed viral Gag-Tax fusion protein, which are significant characteristics of MT-2 cells. Jurkat was obtained within six months of this study from ATCC, U.S.A, who authenticated this cell line by short tandem repeat (STR) analysis. HEK293FT was obtained more than six months before this study from Riken Cell Bank, Japan, who conducted a series of authentication analyses on this cell line, including growth-curve analysis, cell adhesion analysis, isozyme analysis, and STR analysis. Just after arrival to our laboratory, HEK293FT cells were cultured for several days in the conditioned medium and divided to 20 cryo-tubes as live cell-stock, then stored in liquid N_2_ until use. MT-1, HUT102, CEM, Molt-4, HeLa, and HEK293T cells were gifted by the Japanese Foundation for Cancer Research (JFCR) more than 6 months before this study. We authenticated these cell lines by karyotype analysis within 6 months of this study.

**Preparation of primary cells**

For isolation of PBMC, peripheral blood (10 mL) of ATL patients, HTLV-1 asymptomatic carriers, or healthy donors was layered on an equal volume of Ficoll Paque Plus (GE Healthcare, LLC.) and centrifuged at 1,500 rpm for 30 minutes at room temperature. The separated intermediate layer was recovered and suspended in 2 volumes of PBS, and then centrifuged at room temperature at 1,500 rpm for 5 minutes. The supernatant was removed, and cells were suspended in 1 mL of RPMI (Gibco, Thermo Fisher Scientific, Inc.). The cell number was counted in each sample prior to any experiments.

**Construction of protein expression plasmids**

*Human Wnt5a expression plasmids*

The *WT-WNT5A* cDNA fragment was amplified by PCR from the cDNA library prepared from MT-2 cells using the following primers. All primer sequences in the present study are shown in the 5'-to-3' direction. The *ΔE4-WNT5A* cDNA fragment was also amplified by PCR using the following primers from the cDNA library prepared from PBMCs of an acute-type ATL patient. Platinum Taq High Fidelity (Invitrogen, Thermo Fisher Scientific, Inc.) was used for those PCRs. Each amplified cDNA was TA-cloned into the pGEM-T-Easy vector (Promega, Corp.), and the sequence was confirmed. Then, they were amplified by bacteria (*E. coli* strain, DH5α), and extracted using the plasmid extraction kit (Sigma-Aldrich, Merck KGaA). Subsequently, the target *WNT5A* cDNA fragment was excised with EcoRI and XhoI and subcloned into the pCDNA6B (Myc / His) vector (Invitrogen, Thermo Fisher Scientific, Inc.) to add a 6×His tag to the C-terminal of Wnt5a. In order to create a NanoLuc reporter plasmid, the NanoLuc region was excised from the pNL1.1 vector (Promega, Corp.) and inserted into WT-Wnt5a-pCDNA6B and the ΔC-Wnt5a-pCDNA6B at the XhoI and XbaI sites. As a result, Wnt5a with NanoLuc at the C-terminal is expressed. Besides, as a positive control for the NanoLuc assay, a NanoLuc expression plasmid was prepared by inserting a CMV promoter into the multi-cloning site of pNL1.1 vector (Promega, Corp.).

*WT-WNT5A*-For: GAATTCGCCATGAAGAAGTCCATTGG

*WT-WNT5A* -Rev: CTCGAGCGCTTGCACACAAACTGGTCC

*ΔE4-WNT5A*-For: GAATTCGCCATGAAGAAGTCCATTGG

*ΔE4-WNT5A*-Rev: CTCGAGCGGCCAGGTTGTACACCG

*Human FoxM1 expression plasmid*

The *FOXM1* cDNA fragment was amplified with the following primers using a cDNA library prepared from HeLa cells as the template. Platinum Taq High Fidelity (Invitrogen, Thermo Fisher Scientific, Inc.) was used for PCR. The amplified *FOXM1* cDNA was TA-cloned into the pGEM-T-Easy vector (Promega, Corp.), sequenced, and amplified by bacteria (*E. coli* strain, DH5α) and extracted using a plasmid extraction kit (Sigma-Aldrich, Merck KGaA). Subsequently, the *FOXM1* cDNA fragment was excised with BamHI and XhoI, then subcloned into pCDNA6B (Myc/His) vector (Invitrogen, Thermo Fisher Scientific, Inc.). Since *FOXM1* cDNA contains a stop codon, no tag is added to FoxM1 expressed from the FoxM1-pCDNA6B. The same FoxM1 cDNA fragment was also subcloned into pCDNA3.1C vector (Invitrogen, Thermo Fisher Scientific, Inc.) at BamHI and XhoI sites to obtain the His-FoxM1 expression plasmid.

*FOXM1*-For: GGATCCATGAAAACTAGCCCCCGTC

*FOXM1*-Rev: GCTCGAGCTACTGTAGCTCAGG

*Human c-Myb expression plasmid*

The human c-Myb expression plasmids (WT-c-Myb-pCDNA3 and c-Myb-9A-pCDNA3) were kindly gifted by Dr. John P. O'Rourke of the University of New Mexico, U.S.A. Those plasmids express c-Myb proteins without any tags. For construction of FLAG-tagged c-Myb expression plasmids, the cDNA of *MYB* or *MYB-*9A was PCR-amplified with WT-c-Myb-pCDNA3 or c-Myb-9A-pCDNA3 as template, respectively. Platinum Taq High Fidelity (Invitrogen, Thermo Fisher Scientific, Inc.) was used for PCR. The amplified *MYB* cDNA or *MYB-9A* cDNA was TA-cloned into the pGEM-T-Easy vector (Promega, Corp.), sequenced, and amplified by bacteria (*E. coli* strain, DH5α) and extracted using a plasmid extraction kit (Sigma-Aldrich, Merck KGaA). The *MYB* or *MYB-9A* cDNA fragment was excised with XhoI and SpeI, then subcloned into the pME-FLAG vector.

*MYB/MYB-9A*-For: CTCGAGGCCCGAAGACCCCGG

*MYB*-Rev: ACTAGTTCACATGACCAGCGTC

*MYB-9A*-Rev: ACTAGTCTACATAGAATCTATAAATTG

**ChIP (chromatin immunoprecipitation) assays against c-Myb and FoxM1**

For ChIP assays against cMyb, FLAG-WT-c-Myb or FLAG-c-Myb-9A was overexpressed in HEK293FT cells by transfecting WT-c-Myb-pME-FLAG or c-Myb-9A-pME-FLAG using PEI (Polyethileneimine). Then, the nuclear extract was immunoprecipitated with anti-FLAG M2 antibody (#F1804, Sigma-Aldrich, Merck KGaA) or a normal mouse IgG (#I5381, Sigma-Aldrich, Merck KGaA) followed by precipitation with Protein G Sepharose 4 Fast Flow (#17061801, Cytiva). Interaction of FLAG-WT-c-Myb and FLAG-c-Myb-9A with *FOXM1* promoter and *WNT5A* promoter was detected by quantitative RT-PCR (q-PCR) using the primers shown below. For ChIP assays against FoxM1, FoxM1-pCDNA-3.1C, or pCDNA-3.1C as a negative control, was transfected to HEK293FT cells by using PEI. Then, His-FoxM1 or His only in the nuclear extract was precipitated with Ni Sepharose High Performance (#17526802, Cytiva). Interaction of His-FoxM1 with *MYB* promoter and *WNT5A* promoter was detected by quantitative RT-PCR (q-PCR) using the primers shown below.

*FOXM1* promoter

*FOXM1*-ChIP-For: GAACCTTGTCTGCCATTGTATC

*FOXM1*-ChIP-Rev: GCCAAGCCTTCGGATATAATAG

*MYB* promoter

*MYB*-ChIP-For: CTCCATTATGTGAGCGGTGAGG

*MYB*-ChIP-Rev: CAACTGTGCCCTCTGGAGACGG

*WNT5A* promoter

*WNT5A*-ChIP-For: GCCGAACCCTAAAATGAAAGGAC

*WNT5A-*ChIP-Rev: TCCGGGAGATGCCGCTGAAAACGC

**Firefly-luciferase based promoter-reporter assays**

Three promoter reporter plasmids, driven by the *MYB*-promoter, the *WNT5A*-promoter, and the *FoxM1*-promoter, were constructed as follows. Using genomic DNA extracted from normal human PBMC as a template, the *MYB* promoter region (-1112 to -17) and the *WNT5A* promoter region (-555 to +565) were amplified by PCR using the following primers. The Q5 High-Fidelity DNA Polymerase (New England Bio Labs, Inc.) was used in the PCRs. The amplified promoter region was inserted into pGL4.10 (Promega, Corp.) to prepare *MYB*-promoter- and *WNT5A-*promoter reporter plasmids.

*MYB*-prom-For: GGTACCGCAGGTGTGCAATCCAGG

*MYB*-prom-Rev: AAGCTTCTCCGCCGAGAGCCGCG

*WNT5A*-prom-For: CTCGAGGAGCCCTTCCGCCGCCGCC

*WNT5A*-prom-Rev: AAGCTTCGGTCAGGAGCAGGGCTGC

Human *FOXM1* promoter plasmid (-1049 to +194) was purchased from Gene Copoeia, Inc. (#HPRM13918-PG02). In this plasmid, the *FOXM1* promoter region (-1049 to +194) is inserted into the pEZX-PG02 vector. The *FOXM1* promoter region was excised with BglII and HindIII and inserted into pGL4.10 vector (Promega, Corp.) to prepare the *FOXM1* promoter-reporter plasmid.

For luciferase-based reporter assays, HEK293FT cells were seeded at the concentration of 2×10^4^ cells/200μL/well in a 48-well culture plate one day before transfection. At 24 h after seeding, 10ng of the promoter-reporter plasmid, 5ng RSV-*Renilla*-luciferase plasmid, and 200ng of effector plasmids, WT-c-Myb-pCDNA3, c-Myb-9A-pCDNA3, or FoxM1-pCDNA6B, were co-transfected to a well by the PEI transfection method. The empty vector (pCDNA3 or pCDNA6B, respectively) was transfected as mock transfection. For the *WNT5A* promoter analysis with c-Myb and FoxM1 co-transfection, 10ng of the *WNT5A*-promoter reporter plasmid, 5ng RSV-*Renilla*-luciferase plasmid, and 200ng each (400ng total) of WT-c-Myb-pCDNA3, c-Myb-9A-pCDNA3, and/or FoxM1-pCDNA6B was transfected for each well. For expression of c-Myb alone or FoxM1 alone, 200ng of pCDNA3 or pCDNA6B, respectively, was co-transfected with c-Myb-pCDNA3 or FoxM1-pCDNA6B to adjust the total amount of effector expression plasmids. At 24 h after transfection, firefly- and *Renilla*-luciferase activities were measured using the Dual Luciferase Assay System (Promega, Corp.) with the Centro LB 960 luminometer (Berthold Technologies, GmbH & Co.KG.). The firefly-luciferase activity was divided by the corresponding *Renilla*-luciferase activity to normalize transfection efficiency.

**Firefly-luciferase based NF-κB activity reporter assay**

To assess the cellular NF-κB activity, we used a firefly-luciferase based NF-κB activity reporter plasmid, in which firefly-luciferase expression is driven by 6×NF-κB binding sites in pGL4.10 (Promega, Corp.). To examine the effect of Wnt5a inhibition on the NF-κB activity, Jurkat and TL-Om1 cells were treated with IWP-2 (0, 0.1, 1, and 10 μM) for 24 h. Cells were then seeded at the concentration of 2×10^4^ cells / 200 μL / well in a 48-well culture plate and transfected with 100 ng the reporter plasmid and 5 ng RSV-*Renilla*-luciferase plasmid by PEI method. To investigate the effect of the WT-Wnt5a and the ΔC-Wnt5a on the NF-κB activity, HEK293FT cells were seeded at the concentration of 2×10^4^ cells / 200 μL / well in a 48-well culture plate one day before transfection. At 24 h after seeding, 10 ng reporter plasmid, 5 ng RSV-*Renilla*-luciferase plasmid, and 200 ng WT-Wnt5a-pCDNA6B or ΔC-Wnt5a-pCDNA6B were co-transfected by PEI method to the cells in each well. For all assays, firefly-luciferase and *Rennila*-luciferase activities were measured using the dual luciferase assay system (Promega, Corp.) with the Centro LB 960 luminometer (Berthold Technologies GmbH & Co. KG) at 24 h after transfection. Detected firefly-luciferase activity was divided by corresponding *Renilla*-luciferase activity to normalize transfection efficiency.

**Measurement of mean cellular velocity**

For observation of live-cell movement under fluorescent microscope, His-tagged Wnt5a was overexpressed in HEK293FT cells, together with the pCMV-LifeAct-TagGFP2 plasmid (ibidi GmbH). The Lifeact specifically binds to polymerized actin. Therefore, it is suitable to visualize cell movement. Immunocytochemistry was conducted with anti-His antibody (Clone 27E8, #2366S, Cell Signaling Technology, Inc.) and goat anti-mouse IgG conjugated with Alexa Fluor546 (#A-11030, Molecular Probes, Thermo Fisher Scientific, Inc.) to observe the subcellular localization of Wnt5a-His proteins. We observed the movement of the cells overexpressing the WT-Wnt5a or the ΔC-Wnt5a by live-cell imaging with BioStation IM (Nikon, Corp.) and calculated the mean velocity of each cell using ADAPT: Automated Detection and Analysis of ProTrusions^49^.

**Wound-healing assay**

To conduct the wound-healing assay, the WT-Wnt5a-His or the ΔC-Wnt5a-His was overexpressed in HEK293FT cells in a 48-well culture plate at 100% confluent. A scratch was made by the tip of a pipette-tip in each well (wound). Then the images during the wound closure were collected every day for up to 3 days. The size of the cell-covered area was calculated by ImageJ software (Wayne Rasband, NIH, USA), and the percentage of the cell-covered area was calculated as the wound -healing rate (%).

**CXCR4 expression levels**

To measure the CXCR4 expression level on the cell surface by flow-cytometry, 5×10^5^ cells each of Jurkat, CEM, TL-Om1, MT-1, MT-2, and PBMC from an acute-type ATL patient were incubated with anti-CXCR4 antibody conjugated with PE (#555974, BD Biosciences) at the concentration of 100-times dilution in FACS buffer (PBS containing 2% FBS) for 20min at room temperature. Cells were washed in 500 mL of FACS buffer and centrifuged-down at 1,500rpm for 1min. Cells were resuspended in 300 mL of FACS buffer and analyzed by Novocyte Advanteon Flow Cytometer System (Agilent Technologies Inc.). Data were analyzed by FlowJo software (FlowJo LLC).

**The CXCR4/CXCL12-dependent chemotaxis assay**

HEK293FT cells were seeded in a 10 cm dish at a concentration of 4×10^5^ cells / mL. Twenty-four hours later, WT-Wnt5a-pCDNA6B, ΔC-Wnt5a-pCDNA6B, or the empty vector (pCDNA6B) was introduced by the PEI transfection method. The culture supernatant was collected after 48 h as the Wnt5a containing medium. Next, T-cell lines (CEM, Jurkat, MT-2, and TL-Om1) were incubated for 16 h in the culture medium containing the WT-Wnt5a or the ΔC-Wnt5a. The cells were seeded the Wnt5a containing medium in the top chamber of 96-well BME-Coated Cell Invasion Optimization Assay Kit (Trevigen, Inc.) or Transwell with polycarbonate membrane (pore size 8.0 μM) (Corning Inc.) at 2x10^5^ cells / 100 μL / well. The medium with recombinant-CXCL12 (Pepro Tech, Inc.) at 100ng / mL was applied to the bottom chamber at 150 μL / well. After 2.5 h, the numbers of the cells in the bottom chamber (migrated) and in the top chamber (not-migrated) were measured by the Cell Counting Kit (Dojindo Molecular Technologies, Inc.). The cell migration rate was calculated as % [migrated cell number / total cell number (sum of top and bottom cell numbers)]. In the assay, the culture medium from the mock-transfected HEK293FT cells with the empty vector was used as a negative control, while DMEM with rWnt5a (R&D Systems, Inc.) at 100ng / mL was used as a positive control. The CXCR4 antagonist IV (TF14016) was purchased from Calbiochem, Merck Millipore, KGaA (#500507).

**The Wnt5a specific knockdown**

For constructions of a lentiviral plasmid encoding the *WNT5A-*specific shRNA, the sense- and antisense-DNA oligos targeting the *WNT5A* mRNA were annealed by boiling for 5 min followed by cooling-down at *room* temperature for more than 1 h. Annealed fragments were inserted at BglII / XbaI in the pENT4-H1 vector (Invitrogen, Thermo Fisher Scientific, Inc.). Three shRNA expression plasmids were prepared, and two of them (#1 and #2 shown below) showing the best knockdown efficiencies of Wnt5a were used in the experiment.

For the preparation of recombinant lentivirus, HEK293FT cells were seeded at 1x10^5^ cells / mL and 10 mL / 10 cm dish. At 24 h after seeding, 5 μg sh*WNT5A* -CS-RfA-EvBsd, 2.5 μg CAG-HIV-GP, and 2.5 μg pCMV-VSV-G / RSV-Rev were co-transfected by PEI method. At 72 h after transfection, the culture medium was filtrated through 45 μm filter, and the recombinant lentivirus were precipitated by centrifugation at 10,000 rpm for 3 h at 4 ℃. Precipitated viruses were resuspended in 100 μL of RPMI without FBS and stored at -80 ℃ until use.

For transduction of shRNA by infection of recombinant lentivirus, 2x10^6^ cells of Jurkat, MT-2, and TL-Om1 were resuspended in 100 μL sh*WNT5A-*lentivirus stock and processed for centrifugation at 2,000 rpm for 3 h at 35 ℃ in order to enhance infection. After centrifugation, cells were resuspended in 2 mL of RPMI with 10% FBS and incubated at 37 ℃ with 5% CO_2_ for 72 h before used for experiments. In order to examine the effect of Wnt5a knockdown on cell proliferation, the ratio of knockdown cells, i.e., Venus (+) cells, was measured every 3 days for 25 days using a flow cytometer (Novocyte Advanteon Flow Cytometer System, Agilent Technologies Inc.).

sh*WNT5A-* #1

Sense: GATCCCCGTGGGCCGGTTTGTGTGTAAACGTGTGCTGTCCGTTTGCACACAAACTGGTCCACTTTTTGGAAAT

Antisense: CTAGATTTCCAAAAAGTGGACCAGTTTGTGTGCAAACGGACAGCACACGTTTACACACAAACCGGCCCACGGG

sh*WNT5A-* #2

Sense: GATCCCCACATGTAGTACGTCGGGGAAACGTGTGCTGTCCGTTTCTCCGATGTACTGCATGTTTTTTGGAAAT

Antisense: CTAGATTTCCAAAAAACATGCAGTACATCGGAGAAACGGACAGCACACGTTTCCCCGACGTACTACATGTGGG

**Conditional knock-in of Wnt5a by a Tet-on system**

A doxycycline-dependent Wnt5a Tet-on expression was performed by using pRetroX-Tet-On system (Chlontech, Takara Bio Inc.). Briefly, retrovirus encoding rtTA-Advanced, a tetracycline (Tet)-controlled transactivator protein, was prepared by transfecting pRetroX-Tet-On Advanced vector (#PT3968-5-632104, Chlontech, Takara Bio Inc.) with the MuLV-env and MuLV-gag / pol plasmids to the HEK293FT cells by the PEI method. After incubation at 37°C with 5% CO_2_ for 48 h, the supernatant was collected and filtered through a 0.45μm disposable syringe filter (#2053-025, ASAHI GLASS Co. Ltd.) to obtain the retroviral solution. The infection of the retrovirus to TL-Om1 cells were conducted by incubating the 1×10^6^ cells at 37°C with 5% CO_2_ in 2 mL of the medium containing the 1mL viral solution, 1mL RPMI with 10% FBS, and polyburane at 8μg / mL. At 48h, G418 (#A1720, Sigma-Aldrich, Merck KGaA) was added to the culture medium at the concentration of 100 μg / mL, followed by further incubation for 12 days to obtain TL-Om1 cells stably expressing rtTA-Advanced (TL-Om1-Adv). The Tet-responsive Wnt5a expressing retroviral vector was constructed by subcloning the ORF of the WT-Wnt5a or the ΔC-Wnt5a into the pRetroX-Tight-Pur vector (#PT3960-5-632104 Chlontech, Takara Bio Inc.). The pRetroX-Tight-Pur vector, pRetroX-Tight-Pur-WT-Wnt5a, or pRetroX-Tight-Pur-ΔC-Wnt5a, together with the MuLV-env and MuLV-gag / pol plasmids, was transfected to HEK293FT cells by the PEI method to obtain retrovirus solutions of mock, the WT-Wnt5a, or the ΔC-Wnt5a, respectively. TL-Om1-Adv cells were then infected with those retroviruses to obtain TL-Om1-Adv cells with the Tet-responsive gene encoding the WT-Wnt5a, the ΔC-Wnt5a, or no protein (mock). At 48 h after infection, puromycin (#P9620, Sigma-Aldrich, Merck KGaA) was added at 0.5 μg / mL together with G418 at 100 μg / mL followed by further incubation for 12 days to obtain TL-Om1 cells stably expressing rtTA-Advanced together with the Tet-responsive gene encoding the WT-Wnt5a, the ΔC-Wnt5a, or no protein, which are named TL-Om1-Tet-on-WT, TL-Om1-Tet-on-ΔC, or TL-Om1-Tet-on-mock, respectively.

For Wnt5a recovery assay, TL-Om1-Tet-on-WT, TL-Om1-Tet-on--ΔC, and TL-Om1-Tet-on-mock were infected with the lentivirus encoding sh*WNT5A*-#2 to knockdown the endogenous Wnt5a or with the lentivirus encoding sh*LUC* as the negative control. After 14 days, doxycycline-hydrochloride (#D3447, Sigma-Aldrich, Merck KGaA) at 1μg / mL was added to the culture medium to induce Wnt5a expression (ON). Treatment with DMSO at 1μL / mL was also conducted as the negative control (OFF). The population size of Venus (+) cells was analyzed by Novocyte Advanteon Flow Cytometer System (Agilent Technologies Inc.) before and after the induction of Wnt5a to examine the isoform specific effects of Wnt5a on the cell growth. Data were analyzed by FlowJo software (FlowJo LLC).

**Effect of Wnt inhibitor (IWP-2) on cell proliferation**

Firstly, the stock solutions of IWP-2 (Enzo Life Sciences, Inc.) at 0.1, 0.5, and 2.5 mM were prepared in DMSO. Next, cell lines (Jurkat, Molt-4, CEM, MT-2, HUT102, TL-Om1) were counted and incubated in the RPMI (+10% FBS) with IWP-2 at the final concentration of 0.1, 0.5, or 2.5 μM at the cellular concentration, of 5×10^3^ cells / 100 μL / well in a 96-well plate. Cells were also incubated in the medium containing only DMSO at 1μL / mL as the negative control (0 μM). The cell viability was measured daily from day 0 to day 4 using Cell Counting Kit-8 (Dojindo Moleculer Technologies, Inc.).

**Semi-quantitative RT-PCR and quantitative RT-PCR (qPCR)**

For reverse-transcription (RT) PCR, total RNA was extracted from the primary cells and cell lines (Jurkat, CEM, Molt-4, MT-2, C91/PL, HUT102, MT-1, and TL-Om1) with ISOGEN (Nippon Gene Co. Ltd.). Next, cDNA was synthesized from 2 μg of total RNA using SuperScript II (Invitrogen, Thermo Fisher Scientific, Inc.). For the semi-quantitative RT-PCR, PCR was performed with Platinum Taq DNA polymerase (Invitrogen, Thermo Fisher Scientific, Inc.), and the density of the band was examined by electrophoresis on 2% agarose gel. *GAPDH* mRNA was used as an endogenous control. For quantitative-PCR, SYBR Premix Ex Taq (Takara Bio, Inc.) was used, and PCR reaction and quantification were performed by Thermal Cycler Dice Real-Time (Takara Bio, Inc.). As endogenous controls, *β-ACTIN* mRNA levels (for cell lines) and *RPL19* mRNA levels (for primary cells) were used. The primer sequences used in the semi-quantitative RT-PCR and q-PCR are shown below.

Semi-quantitative RT-PCR

*WNT5A (Ex3)-*For: CTGTGCCACTTGTATCAGGACC

*WNT5A (Ex5)-*Rev: GAAGCGGCTGTTGACCTGTACC

*GAPDH*-For: TCGTGGAAGGACTCATGACC

*GAPDH*-Rev: TCCACCACCCTGTTGCTGTA

qPCR

*WNT5A* (qPCR)-For: GGTATATCACATGTCTCATTC

*WNT5A (*qPCR)-Rev: GGACTTTCCTCCCTGCTGCCC

*β-ACTIN* (qPCR)-For: GCCTGACGGCCAGGTCAT

*β-ACTIN* (qPCR)-Rev: CAGGACTCCATGCCCAGGAA

*RPL19* (qPCR)-For: ACCAAGGAAGCACGCAAGC

*RPL19* (qPCR)-Rev: CAGACAAAGTGGGAGGTTTTATTTC

**Western blotting**

To prepare whole cell lysate samples, cells were lysed in RIPA Buffer (0.01 M Tris-HCl (pH 7.4), 1% NP-40, 0.1% Sodium Deoxycholate, 0.1% SDS, 0.15 M NaCl, 0.01 M EDTA), and proteins were separated by SDS-PAGE in 10% or 12% acrylamide gels. Next, proteins were transferred to a PVDF membrane (Merck, KGaA) in a transfer buffer (3.03 mg / L Tris, 14.41 mg / ml Glycine, 20% Methanol, 0.05% Triton X-100) using a wet or a semi-dry blotting method. The membrane was blocked with blocking buffer (5% skim milk in TBST (TBS + 0.5% Tween-20)) for 1 h and incubated in a blocking buffer containing primary antibody at 4 ℃ for overnight. The membrane was washed in TBST (10 min for 3 times) and incubated with a secondary antibody labeled with alkaline phosphatase at room temperature for 1 h. The membrane was washed in TBST (5 min for 3 times), and bands were detected using NBT / BCIP (Promega, Corp.).

For the detection of the endogenous WT-Wnt5a, the anti-Wnt5a rabbit monoclonal antibody (#sc-12100, Cell Signaling Technology, Inc.) was used as the primary antibody. For the detection of the ΔC-Wnt5a, the anti-Wnt5a rabbit polyclonal antibody (#ab-186995, Abcam, Plc.), which has the epitome in the N-terminal region of Wnt5a protein, was used. For detection of The His-tagged Wnt5a overexpressed in HEK293FT cells, the His-tag mouse monoclonal antibody (Clone 27E8, #2366S, Cell Signaling Technology, Inc.) was used. To analyze the activation of the Src signaling pathway in HEK293FT cells overexpressing the WT-Wnt5a or the ΔC-Wnt5a, following Src antibodies were used as primary antibodies; total-Src rabbit monoclonal antibody (Clone 3266, #2123P, Cell Signaling Technology, Inc.), P-Src (Tyr416) rabbit monoclonal antibody (Clone D49G4, #6943P, Cell Signaling Technology, Inc.), and P-Src (Tyr527) rabbit antibody (#2105P, Cell Signaling Technology, Inc.). For the sample-loading control, the β-Actin level was analyzed by the anti β-Actin mouse monoclonal antibody (#sc-69879, Santa Cruz Biotechnology, Inc.). Alkaline-phosphatase (AP)-conjugated goat anti-mouse IgG (S372B, Promega, Corp.) or goat anti-rabbit IgG (S373B, Promega, Corp.) was used as the secondary antibody.

**Supplementary Results**


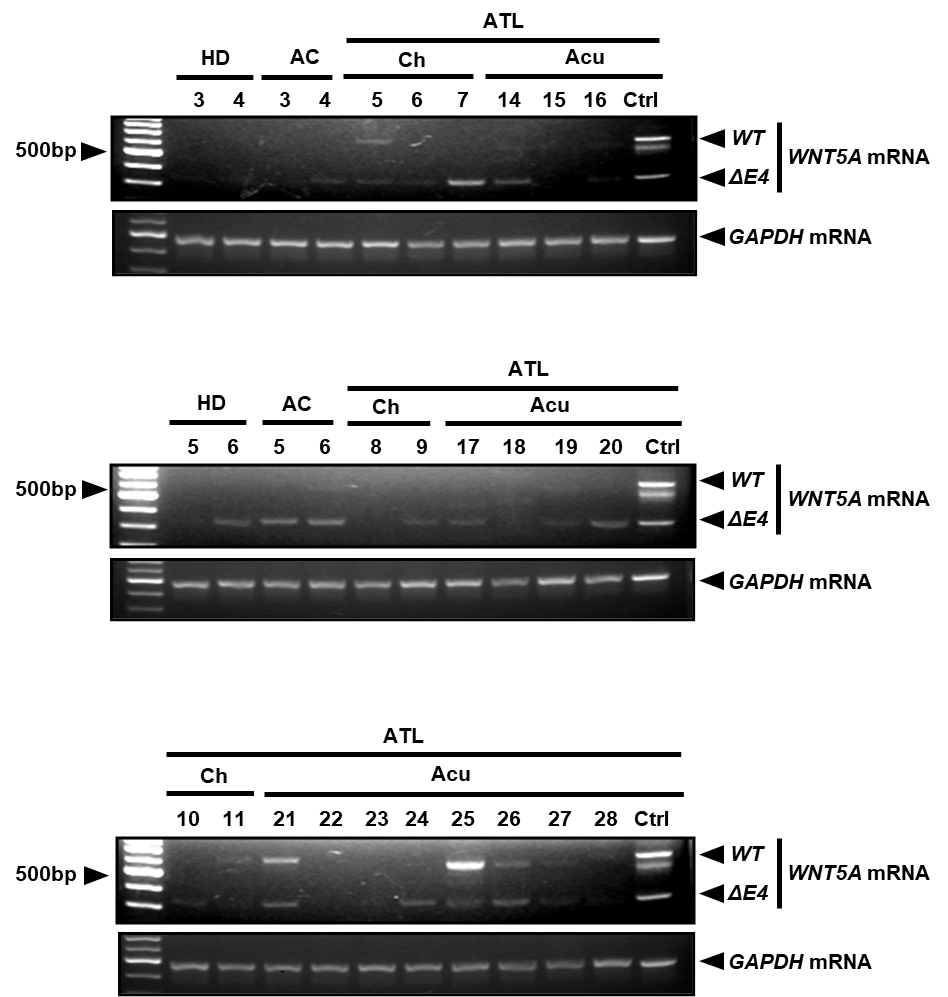


**Figure S1. The semi-quantitative RT-PCR for the *WNT5A* mRNA.**

Total RNA samples extracted from normal CD4^+^ T cells (n = 6), HTLV-1 asymptomatic carriers (ACs; n = 6), and ATL patients (smoldering type; n = 2, chronic type; n = 10, and acute type; n = 17) were subjected to the semi-quantitative RT-PCR with the primers at the exon3 and exon5 of human *WNT5A* mRNA. These primers amplify 630bp fragment for the *WT-WNT5A* mRNA and 293bp fragment for the *WNT5A* mRNA without the exon4 (the *ΔE4-WNT5A* mRNA). Please note that each panel consists of two pictures from two separated agarose gels for *WNT5A* mRNA and *GAPDH* mRNA, respectively, which are clearly separated by black frames. This figure is related to Figure 4.

**
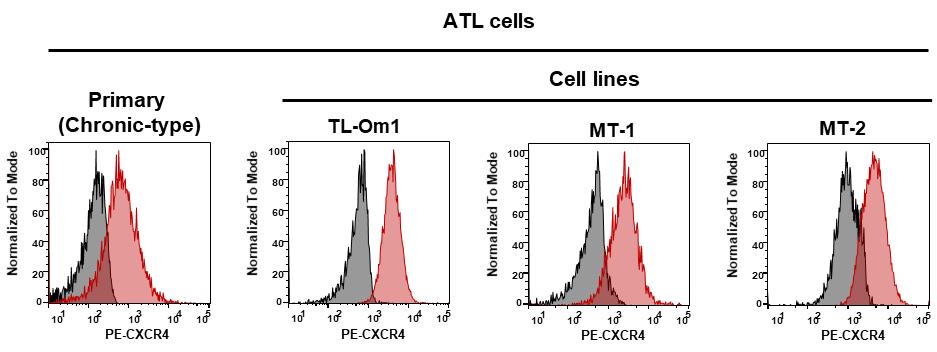
**

**Figure S2. CXCR4 levels in ATL cells**

CXCR4 expression levels were analyzed in primary ATL cells (PBMCs from a chronic-type ATL patient-#29) and in HTLV-1 related cell lines (TL-Om1, MT-1, and MT-2). CXCR4 is overexpressed in primary ATL cells in a similar magnitude as ATL-patient derived cell lines (TL-Om1 and MT-1) and a HTLV-1 immortalized cell line (MT-2). This figure is related to Figure 7A.

**A B**

**WT-Wnt5a**

**∆C-Wnt5a**


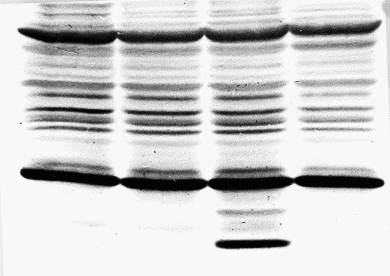

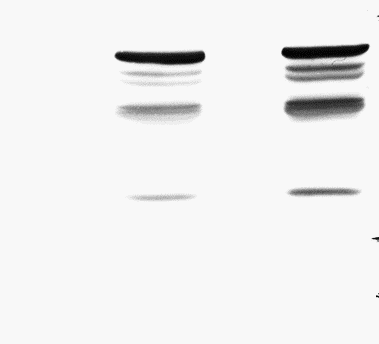

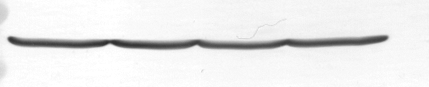

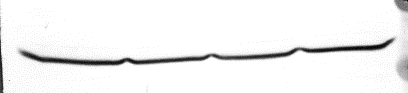


**Total Src**

**P-Src (Tyr416)**

**P-Src (Tyr527)**


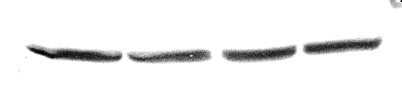


**β-Actin**

**WT-Wnt5a**


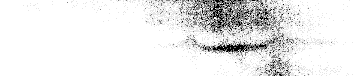


**Mock**

**∆C-Wnt5a**


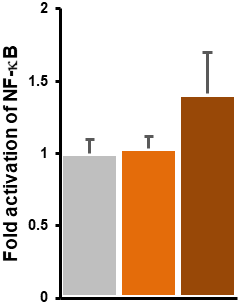


**WT-Wnt5a**

**∆C-Wnt5a**

**Mock**

***

**Figure S3. The ΔC-Wnt5a activates the NF-κB and the Src pathways**

**A.** The luciferase-based reporter assays showed that the NF-κB activity was significantly elevated in the ΔC-Wnt5a-expressing HEK293FT cells compared with the mock-transfected control cells. The WT-Wnt5a did not activate NF-κB in HEK293FT cells (n = 6, Mean±SD, ****P*<0.001). **B.** The levels of the activated Src (phosphorylated at Tyr416) and the non-activated Src (phosphorylated at Tyr527) were examined by Western blotting of HEK293FT cell lysate overexpressing the WT-Wnt5a or the ΔC-Wnt5a. The results showed that the levels of the total Src and the P-Src (Tyr527) did not change significantly among cells. On the other hand, the band of the P-Src (Tyr416) was detected only in the ΔC-Wnt5a-expressing cells. Please note that this figure is consists of six pictures from six separated blots for WT-Wnt5a, ΔC-Wnt5a, total Src, P-Src (Tyr527), P-Src (Tyr416), and β-Actin, respectively, which are clearly separated by black frames. This figure is related to Figure 7.

**
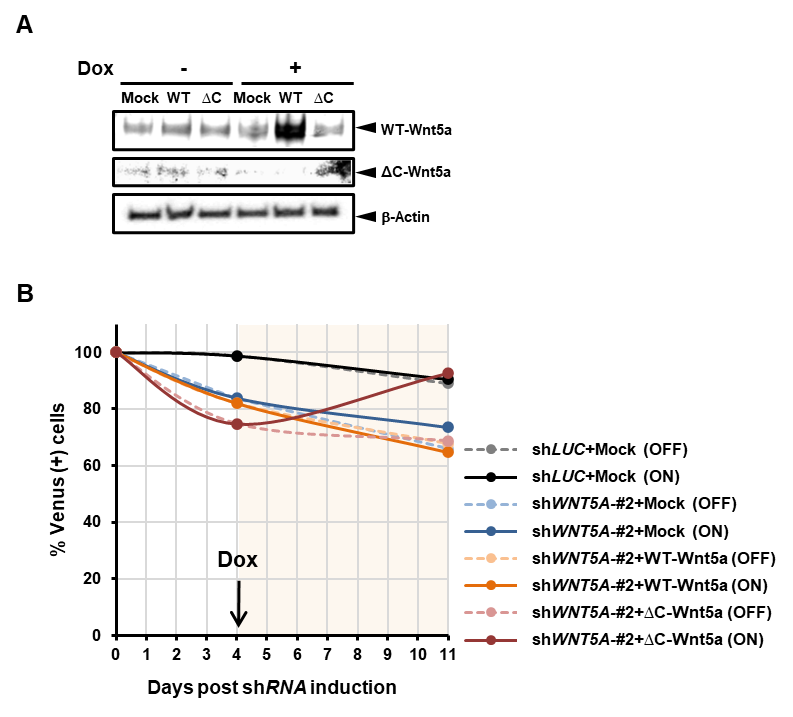
**

**Figure S4. Wnt5a isoform-specific effects on the cell viability**

**A.** TL-Om1-Tet-on-WT or TL-Om1-Tet-on-ΔC cells express the WT-Wnt5a or the ΔC-Wnt5a, respectively, while the TL-Om1-Tet-on-mock cells do not, by a doxycycline treatment. **B.** The graphs show the changes in the percentage of Venus (+) cells, i.e., Wnt5a knockdown in TL-Om1-Tet-on-Wnt5a / mock cells. The cell growth rate is recovered only in TL-Om1-Tet-on-ΔC cells after a doxycycline treatment (ON). The mock treatment with DMSO was also conducted (OFF). This figure is related to Figure 7B.

**Supplementary Table**

**Table S1. Information of primary samples**

**Original Western blotting images**

**
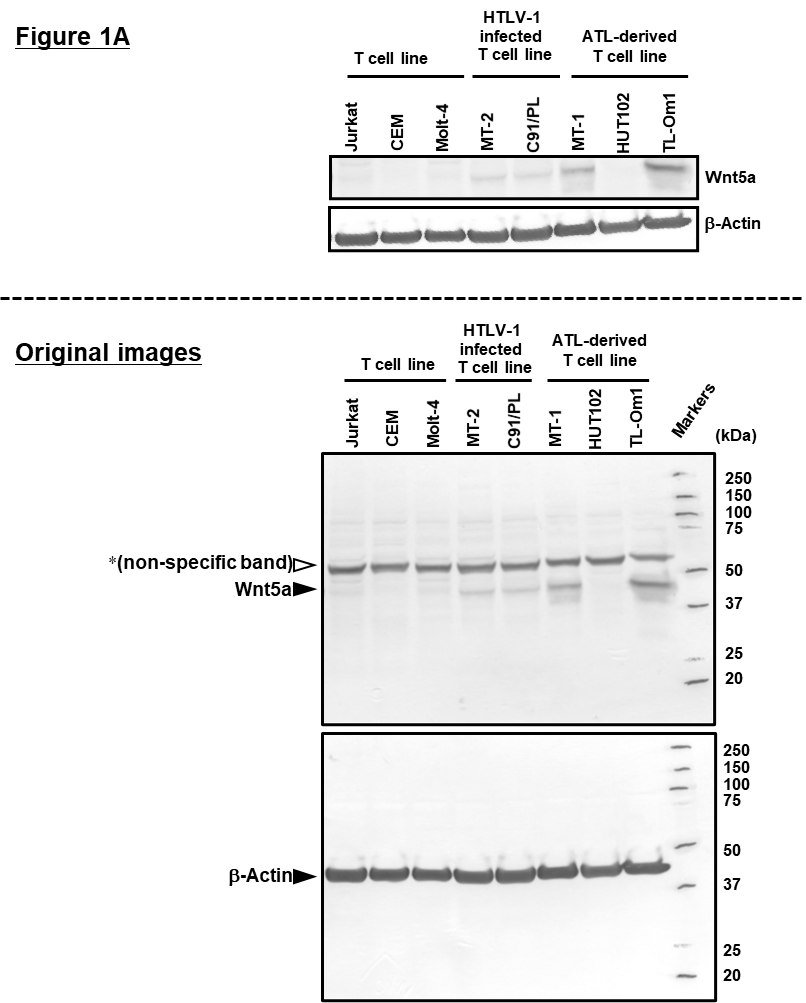
**

**Figure S5. Original Western blotting images of Figure 1A**

The original images of the Western blotting membranes (below), which are shown in Figure 1A (top). In each image, almost the entire area, which is related to the present study, is shown.

**
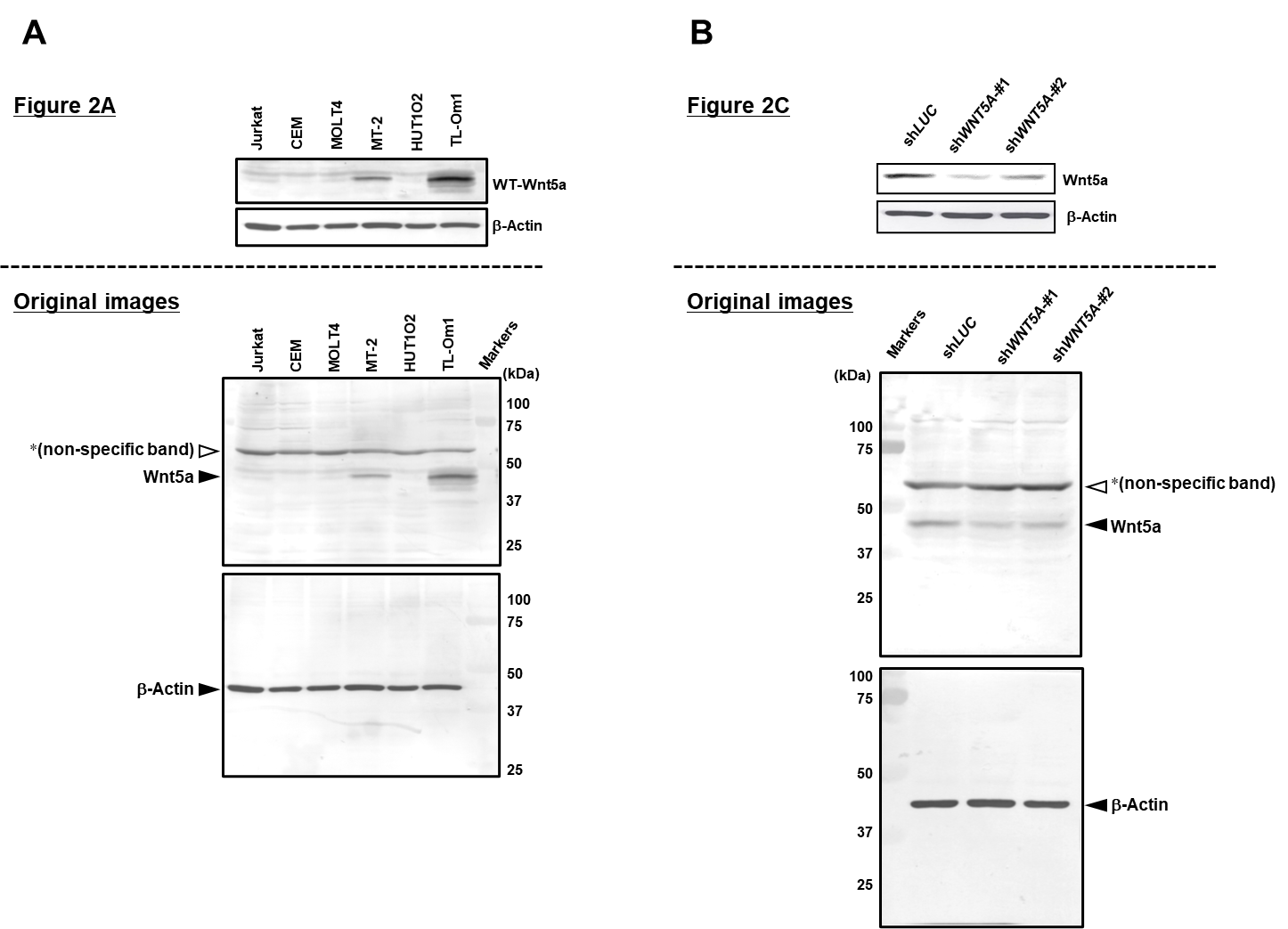
**

**Figure S6. Original Western blotting images of Figure 2A and 2C**

**A.** The original images of the Western blotting membranes (below), which are shown in Figure 2A (top). **B.** The original images of the Western blotting membranes (below), which are shown in Figure 2C (top). In each image, almost the entire area, which is related to the present study, is shown.


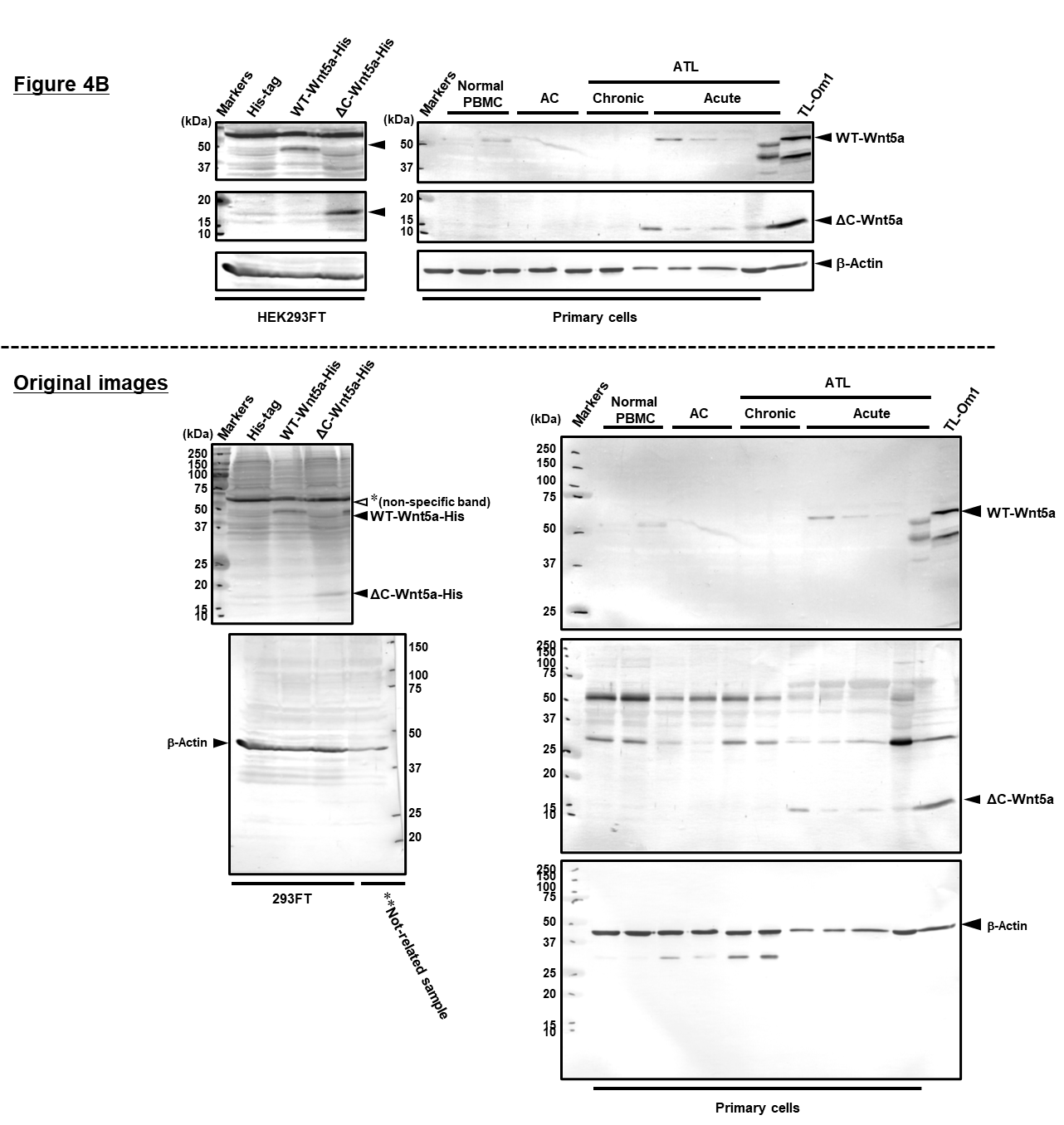


**Figure S7. Original Western blotting images of Figure 4B**

The original images of the Western blotting membranes (below), which are shown in Figure 4B (top). In each image, almost the entire area, which is related to the present study, is shown.

**
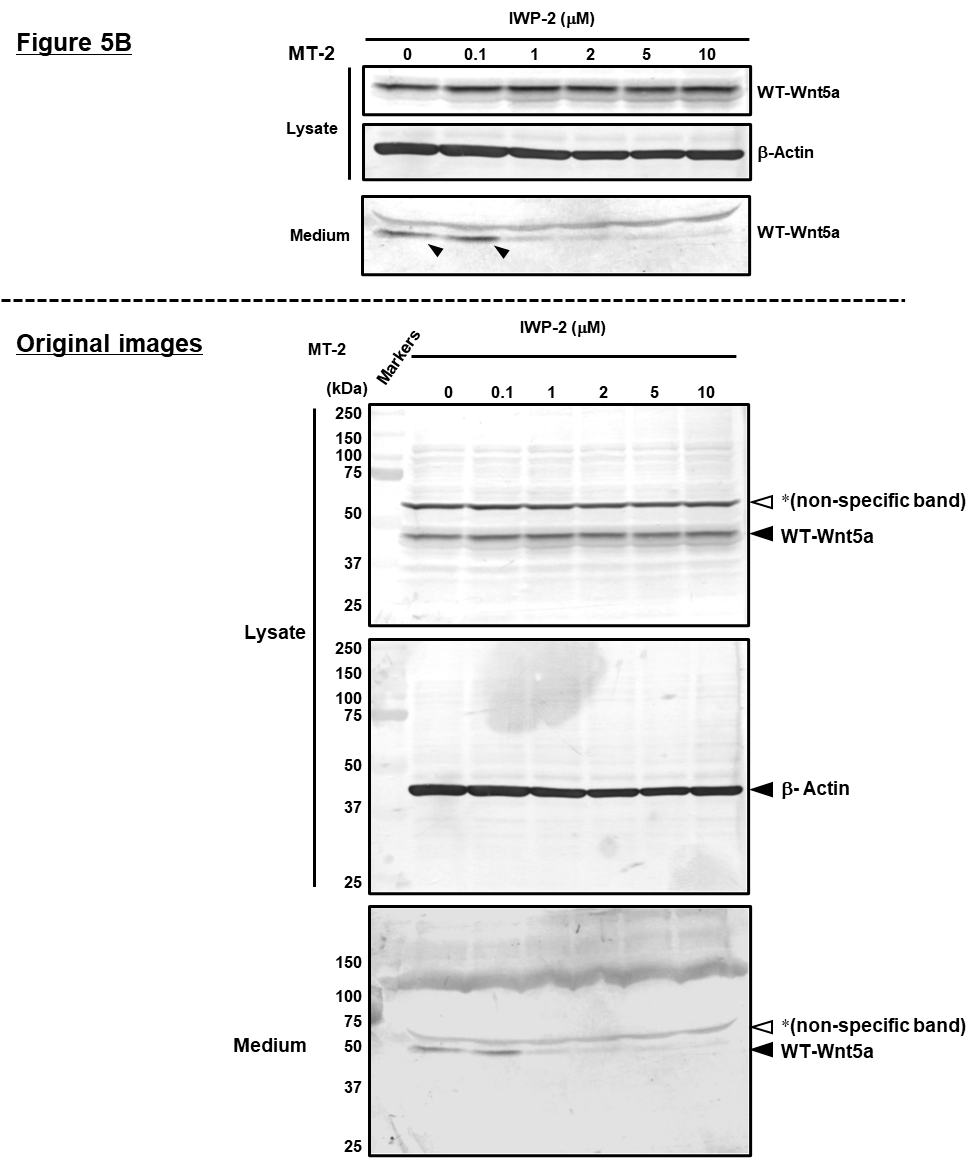
**

**Figure S8. Original Western blotting images of Figure 5B**

The original images of the Western blotting membranes (below), which are shown in Figure 5B (top). In each image, almost the entire area, which is related to the present study, is shown.
